# Stress-induced TRBP phosphorylation enhances its interaction with PKR to regulate cellular survival

**DOI:** 10.1038/s41598-018-19360-8

**Published:** 2018-01-18

**Authors:** Evelyn Chukwurah, Rekha C. Patel

**Affiliations:** Department of Biological Sciences, University of South Carolina, Columbia, SC 29208 USA

## Abstract

Transactivation response element RNA-binding protein (TRBP or TARBP2) initially identified to play an important role in human immunodeficiency virus (HIV) replication also has emerged as a regulator of microRNA biogenesis. In addition, TRBP functions in signaling pathways by negatively regulating the interferon-induced double-stranded RNA (dsRNA)-activated protein kinase (PKR) during viral infections and cell stress. During cellular stress, PKR is activated and phosphorylates the α subunit of the eukaryotic translation factor eIF2, leading to the cessation of general protein synthesis. TRBP inhibits PKR activity by direct interaction as well as by binding to PKR’s two known activators, dsRNA and PACT, thus preventing their interaction with PKR. In this study, we demonstrate for the first time that TRBP is phosphorylated in response to oxidative stress and upon phosphorylation, inhibits PKR more efficiently promoting cell survival. These results establish that PKR regulation through stress-induced TRBP phosphorylation is an important mechanism ensuring cellular recovery and preventing apoptosis due to sustained PKR activation.

## Introduction

The double-stranded RNA (dsRNA)-activated protein kinase (PKR) is an interferon (IFN)-induced serine/threonine protein kinase expressed ubiquitously in mammalian cells^1–3^. Although IFNs induce expression of PKR at a transcriptional level, PKR’s kinase activity stays latent until it binds to one of its activators leading to its autophosphorylation and catalytic activation^4^. The best-characterized cellular substrate of PKR is the translation initiation factor, eIF2α, the phosphorylation of which on serine 51 (S51) results in an inhibition of protein synthesis^5,6^. An immediate response of cells exposed to various forms of stress is a general inhibition of protein synthesis, which is mainly caused by the increased S51 phosphorylation of eIF2α^7^. The eIF2α phosphorylation thus serves an important function to block the general protein synthesis and allow cells to either recover from stress or undergo apoptosis when damage is beyond repair^8^. PKR plays an important role in regulating apoptosis after exposure to several diverse stress signals that include viral pathogens, oxidative stress, endoplasmic reticulum (ER) stress, and growth factor or serum deprivation^9,10^.

During viral infections, the double-stranded (ds) RNA, which is a replication intermediate for several viruses^11^, activates PKR by a direct interaction. The dsRNA binds to PKR via the two dsRNA-binding motifs (dsRBMs) present at the N terminus^12–15^, changing the conformation of PKR to expose its ATP-binding site^16,17^ and consequent autophosphorylation^18^. The two dsRBMs also mediate dsRNA-independent protein-protein interactions with other proteins that carry similar domains^19,20^. Among these are proteins inhibitory for PKR activity such as **T**AR **R**NA-**b**inding **p**rotein (TRBP)^21^, and also a **P**KR **act**ivating protein (PACT)^22,23^. PKR activation in response to stress signals is tightly regulated by PACT and TRBP, both acting to regulate its catalytic activity by a direct interaction with PKR as well as with each other^24,25^. As the dsRBMs in PKR, PACT, and TRBP mediate protein-protein interactions^26^, these three proteins form both heterodimers as well as homodimers and the stress-dependent phosphorylation of PACT changes the relative strengths of PKR-PACT, PACT-TRBP, and PACT-PACT interactions to bring about a timely and transient PKR activation with precise control^25,27^. This regulates the general kinetics as well as level of eIF2α phosphorylation thereby influencing the cellular response to stress either as recovery and survival or elimination by apoptosis^28^.

TRBP has three dsRBMs; the first two are true dsRBMs and interact with dsRNA, while the third carboxy-terminal dsRBM mediates TRBP’s interactions with other proteins such as Dicer, and Merlin^26,29,30^. TRBP inhibits PKR by interacting with dsRNA and sequestering it away from PKR as well as by forming PKR-TRBP heterodimers^21,31^. In the absence of viral infections and stress signals, TRBP forms heterodimers with both PKR and PACT, preventing their association and PACT-mediated PKR activation^24,32^. Importantly, the stress-induced serine 287 phosphorylation of PACT decreases its interaction with PKR inhibitory protein TRBP thereby further aiding in rapid PKR activation following exposure to stress signals^24,25^. In contrast, not much is known about how similar post-translational modifications may affect TRBP’s interaction with PKR and consequently, its ability to inhibit PKR during cellular stress. Previous reports indicate that TRBP is phosphorylated by the two MAPKs; ERK 1/2 and JNK, with specific effects on RISC component stability and PKR activation by endogenous *Alu* transcripts during mitosis respectively^33,34^. In this study, we used various biochemical assays to determine if TRBP undergoes stress-induced phosphorylation, and if this affects TRBP’s ability to inhibit PKR during oxidative stress. Our findings implicate MAPKs (ERK1/2 and JNK) in oxidative stress-induced TRBP phosphorylation, and show that TRBP phosphorylation significantly enhances TRBP’s ability to interact with and inhibit PKR during oxidative stress to regulate apoptosis.

## Results

### TRBP overexpression inhibits oxidative stress-induced apoptosis

**Figure 1:**
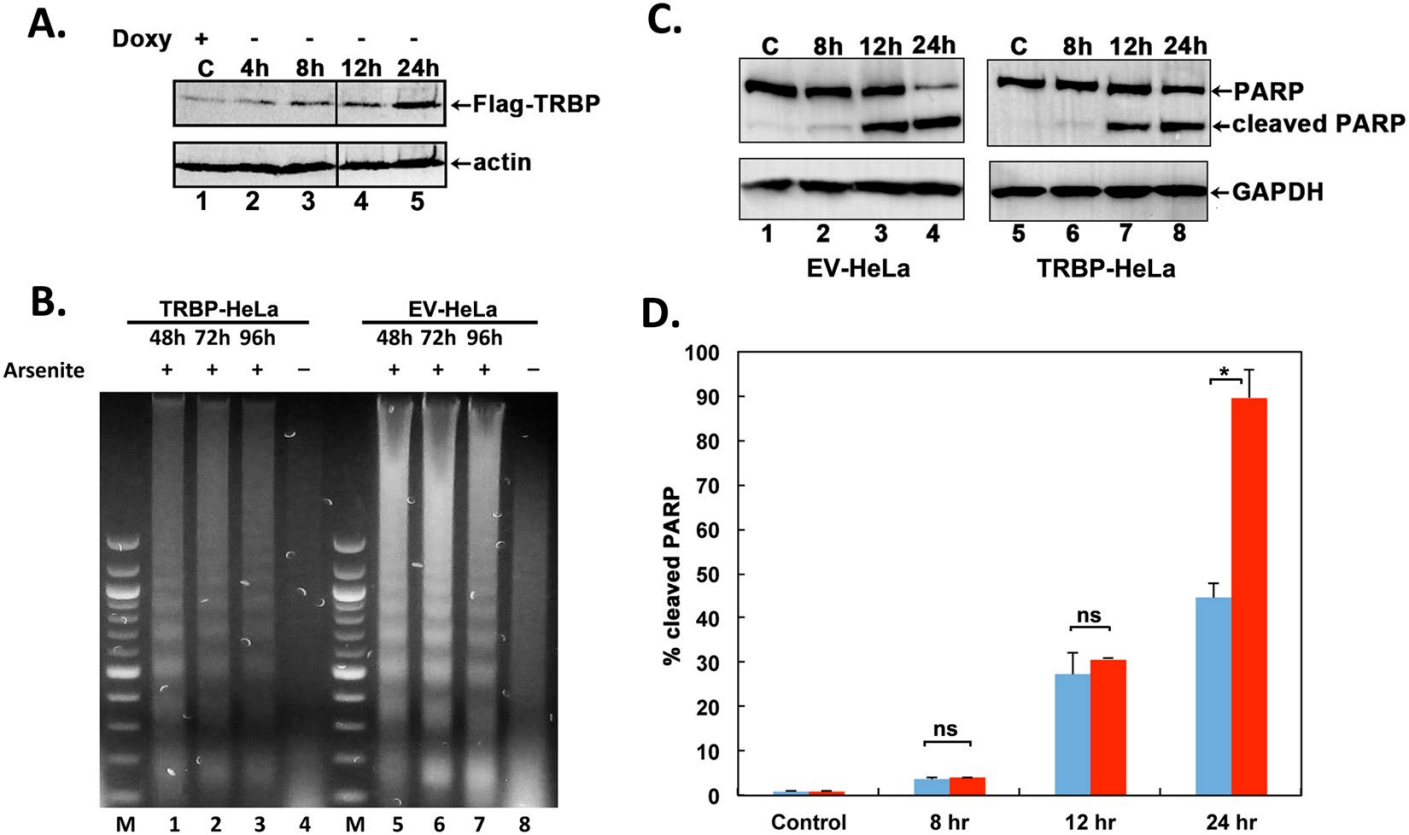
TRBP overexpression protects cells from arsenite-induced apoptosis. (**A**) Establishment of a stable doxycycline-inducible HeLa cell line overexpressing flag-TRBP. HeLa-Tet off (Clontech) cells were transfected with either FlagTRBP/pTRE2pur expression construct or pTRE2pur (Clontech) empty vector (EV). Puromycin resistant clones were isolated, characterized, and one clone (TRBP-HeLa) that showed inducible expression of FlagTRBP was selected for further studies. Induction of FlagTRBP expression after removal of doxycycline from the growth medium at indicated time points is shown. Western blot analysis of cell lysates using 50 μg of total protein was performed using anti-Flag and anti-β-actin antibodies. The black line between lanes 3 and 4 represents where different lanes from the same western blot were joined. (**B**) DNA Fragmentation Analysis. TRBP-HeLa cells overexpressing FlagTRBP (Lanes 1–4) or EV-HeLa control cells (Lanes 5–8) were treated with 10 μM sodium arsenite for 48, 72, and 96 hours, fragmented DNA was isolated and analyzed. M: 100-bp ladder, Lanes 1 & 5: 48 hr treatment. Lanes 2 & 6: 72 hr treatment, Lanes 3 & 7: 96 hr treatment, and Lanes 4 & 8: untreated cells. (**C**) Analysis of PARP cleavage in response to arsenite treatment. TRBP-HeLa and EV-HeLa cells were treated with 25 μM sodium arsenite for the indicated time points, and western blot analysis using anti-PARP antibody was performed on cell lysates containing 50 μg of total protein to assess increases in poly ADP-ribose polymerase (PARP1) cleavage. Western blot was also performed with anti-GAPDH antibody to ensure equal protein in all samples. (**D**) Quantification of PARP cleavage. PARP1 and cleaved PARP1 bands were quantified using ImageQuant TL Software. The percentage of cleaved PARP1 was calculated as (cleaved PARP1 band intensity/cleaved + uncleaved bands intensities) X 100. Bars represent percentages of cleaved PARP1 from 3 independent experiments. Error bars represent standard deviation (S.D.) from three experiments. Student’s t tests were performed to determine statistical significance – ns: not significant, asterisk: p value of 0.000352.

To evaluate TRBP’s effect on the cellular response to oxidative stress, we established a stable HeLa-Tet off cell line that would conditionally overexpress Flag-TRBP only when doxycycline was absent from the growth medium. A HeLa-Tet off cell line with stably transfected empty vector pTRE2pur was established as a control. We initially characterized 20 individual puromycin resistant clones and selected one clone that showed the least expression of Flag-TRBP in the presence of doxycycline and showed a good induction of Flag-TRBP expression in the absence of doxycycline. As seen in Fig. 1A, the Flag-TRBP expression is induced to high levels in a time dependent manner after removal of doxycycline from the growth medium (lanes 2–5). We used these cells for assaying the effects of TRBP overexpression on apoptosis induced by oxidative stress. After the cells were grown in doxycycline-deficient growth medium for 24 h, they were exposed to sodium arsenite to induce oxidative stress. The cells were thus expressing high level of Flag-TRBP when exposed to oxidative stress and this allowed us to assay the effect of TRBP overexpression on cellular apoptosis and PKR activation.

In order to compare the relative apoptosis in control and TRBP overexpressing cells we used DNA fragmentation analysis. DNA fragmentation is a late marker of apoptotic cells as the DNA is cleaved by caspase-activated DNases (CADs) into nucleosomal fragments of 180 bp^35^. As seen in Fig. 1B, the control cells stably transfected with empty vector (EV-HeLa) showed high levels of DNA fragmentation in response to sodium arsenite (lanes 5–8). In comparison, the cells overexpressing Flag-TRBP (TRBP-HeLa) have significantly less DNA fragmentation after exposure to sodium arsenite (lanes 1–4). These results indicate that TRBP overexpressing cells are significantly protected from oxidative stress-induced apoptosis.

In order to further assess the protection from cellular apoptosis by TRBP overexpression, we compared the cleavage of Poly-ADP Ribose Polymerase (PARP1) in response to arsenite. The 116 kDa protein PARP1 is cleaved into an 89 kDa fragment by Caspase-3 in response to apoptosis-inducing stimuli^36^. We measured and quantified PARP1 cleavage in both sets of cells after treatment with arsenite (Fig. 1C,D). As seen in Fig. 1C, there is a steady increase in the levels of cleaved PARP1 in the control (EV-HeLa) and TRBP-overexpressing (TRBP-HeLa) cells in a time dependent manner. After 24 hours of arsenite exposure, there is significantly more cleaved PARP1 in the EV-HeLa cells (Lane 4) as compared to the TRBP-HeLa cells (Lane 8). The percentage of cleaved PARP1 is about 90% in the HeLa cells (Fig. 1D, 24 hr), and only about 45% in the TRBP-HeLa cells (Fig. 1D, 24 hr). These results indicate that caspase-3 activation and subsequent PARP1 cleavage is significantly impaired in cells overexpressing TRBP, and demonstrate that TRBP overexpression protects the cells from apoptosis in response to oxidative stress.

### Both ERK and JNK phosphorylate TRBP in response to oxidative stress

**Figure 2:**
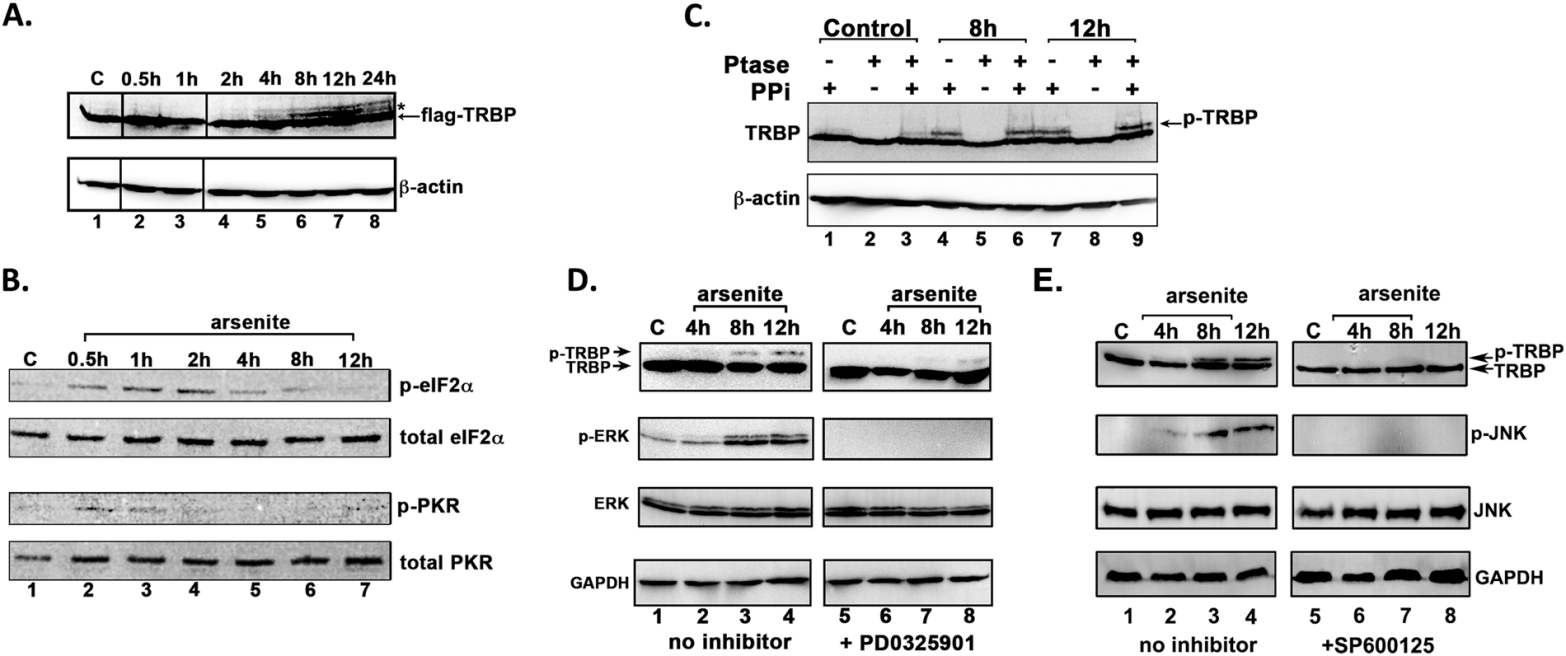
TRBP is phosphorylated by ERK and JNK in response to arsenite-induced oxidative stress. (**A**) TRBP’s electrophoretic mobility shifts in response to sodium arsenite treatment. A western blot analysis of 50 μg protein per lane from HeLa-TRBP cells treated with 10 μM sodium arsenite at the indicated time points is shown. Western blot analysis was performed with anti-Flag and anti-β actin antibodies. The slower migrating TRBP band at 8 h, and 12 h is indicated by an asterisk. The line between lanes 1 and 2 as well as between lanes 3 and 4 represents where lanes from the same western blot were joined. (**B**) PKR phosphorylation and eIF2α phosphorylation kinetics in response to sodium arsenite treatment. HeLa cells were treated with 10 µM sodium arsenite and cell extracts were prepared at the indicated time points. PKR and eIF2α phosphorylation status at each time point was determined by a western blot analysis using anti-phospho-PKR and anti-phospho-eIF2α specific antibodies using 100 µg and 10 µg of total protein respectively. Each blot was subsequently stripped and re-probed with anti- eIF2α or anti-PKR antibody to ensure equal loading in all lanes. (**C**) TRBP is phosphorylated in response to oxidative stress. Extracts from untreated, and 8 h, or 12 h arsenite-treated TRBP-HeLa cells were prepared in the presence or absence of phosphatase inhibitor (PPi) as indicated above the lanes and subsequently treated with phosphatase (Ptase) or left untreated as indicated. Western blot to was performed with anti-Flag antibody followed by anti- β-actin antibody. (**D**) ERK is phosphorylated in response to oxidative stress and phosphorylates TRBP. TRBP overexpressing TRBP-HeLa cells were treated with 10 μM arsenite alone or in combination with the MEK inhibitor PD0325901 for 24 hours. Cell extracts were made at the indicated time points, and western blot analysis was performed using anti-Flag, anti-phospho-ERK, anti-total ERK, and anti-GAPDH antibodies. (**D**) JNK is phosphorylated in response to oxidative stress and phosphorylates TRBP. TRBP overexpressing TRBP-HeLa cells were treated with 10 μM arsenite alone or in combination with the MEK inhibitor SP600125 for 24 hours. Cell extracts were made at the indicated time points, and western blot analysis was performed using anti-Flag, anti-phospho-JNK, anti-total JNK, and anti-GAPDH antibodies.

In order to determine if TRBP undergoes post-translational modifications in response to stress signals with any functional implications on TRBP’s ability to inhibit PKR. Western blot analysis of extracts from TRBP-HeLa cells over 24 hours of exposure to sodium arsenite revealed the presence of an additional Flag-TRBP band with reduced electrophoretic mobility as indicated by an asterisk (Fig. 2A) that increased in intensity from 8 to 12 hours after arsenite treatment and declined at 24 hours after treatment (Fig. 2A, Flag-TRBP panel, Lanes 5–8). These results suggested that the slow migrating Flag-TRBP band may be indicative of TRBP phosphorylation at late time points after arsenite exposure. Interestingly, we also noted that the strengthening of the TRBP doublet banding pattern from 8 to 12 hours after treatment coincides with a decrease in phosphorylated eIF2α levels at these time points after sodium arsenite treatment (Fig. 2B: p-eIF2α panel, Lanes 6–7) and a decrease in phosphorylated PKR levels (Fig. 2B: p-PKR panel, Lanes 5–7). The phospho-eIF2α levels show a decrease at 4 h (Fig. 2B, lane 5) and the phospho-TRBP is also detectable at 4 h (Fig. 2A, lane 5), although the phosphorylation of TRBP continues to rise at 8 and 12 h (Fig. 2A, lanes 6–7), also coinciding with a further decrease in phospho-eIF2α levels (Fig. 2B, lanes 6–7). These results suggest that TRBP phosphorylation may regulate PKR activation and consequent eIF2α phosphorylation in response to arsenite. To test this, we investigated if the slow migrating TRBP band resulted from phosphorylation by using phosphatase treatment in the presence and absence of phosphatase inhibitors. Phosphatase treatment of cell extract prepared 8 and 12 hours after arsenite treatment completely removed the stress-induced slow- migrating band (Fig. 2B, lanes 5 and 8), demonstrating that the slower mobility band (denoted ‘p-TRBP’) did result from TRBP phosphorylation in response to oxidative stress. The p-TRBP band persisted when the phosphatase treatment was performed in the presence of phosphatase inhibitors, thereby confirming that the disappearance of the band in lanes 5 and 8 was indeed due to phosphatase activity and not due to the presence of any contaminating proteolytic activity. These results indicate a possible link between the timing of PKR activation and its eventual inactivation during cell stress and the timing of TRBP phosphorylation in response to oxidative stress.

The **M**itogen-**a**ctivated **p**rotein **k**inase (MAPK) signaling pathways are activated in response to diverse stimuli^37^, and elicit either pro-apoptotic or pro-survival cellular responses. Previous studies have demonstrated that MAPKs such as the **E**xtracellular-signal **r**egulated **K**inase (ERK 1/2) and c-Jun N-terminal kinase (JNK) play important roles in mediating the cellular response to oxidative stress^38^. To test if ERK 1/2 phosphorylates TRBP in response to oxidative stress, we pretreated the Flag TRBP overexpressing cells with the MEK inhibitor PD0325901, and exposed the cells to sodium arsenite. In the samples not pretreated with PD0325901, we observed the p-TRBP band at 8 and 12 hrs after treatment (Fig. 2D: TRBP panel, Lanes 1–4). Furthermore, we also observed that the increase in TRBP phosphorylation closely mirrored the increase in phospho-ERK levels at 8, and 12 hours of treatment (Fig. 2D, p-ERK panel, Lanes 1–4). With the inhibition of ERK phosphorylation, (Fig. 2D, p-ERK panel, Lanes 5–8) the p-TRBP band is significantly diminished (Fig. 2D, TRBP panel, Lanes 5–8). To test if JNK also phosphorylates TRBP in response to oxidative stress, we pretreated the Flag TRBP overexpressing cells with the JNK inhibitor SP600125, and exposed the cells to sodium arsenite. In the samples not pretreated with SP600125, we observed the p-TRBP band at 8 and 12 hrs after treatment (Fig. 2E: TRBP panel, Lanes 1–4). Furthermore, we also observed that the increase in TRBP phosphorylation closely mirrored the increase in phospho-JNK levels at 8, and 12 hours of treatment (Fig. 2E, p-ERK panel, Lanes 1–4). With the inhibition of JNK phosphorylation, (Fig. 2E, p-JNK panel, Lanes 5–8) the p-TRBP band is completely absent (Fig. 2E, TRBP panel, Lanes 5–8). The results in Fig. 2D,E suggest that both ERK and JNK phosphorylate TRBP in response to oxidative stress.

### Effect of TRBP phosphorylation on cellular response to stress

**Figure 3:**
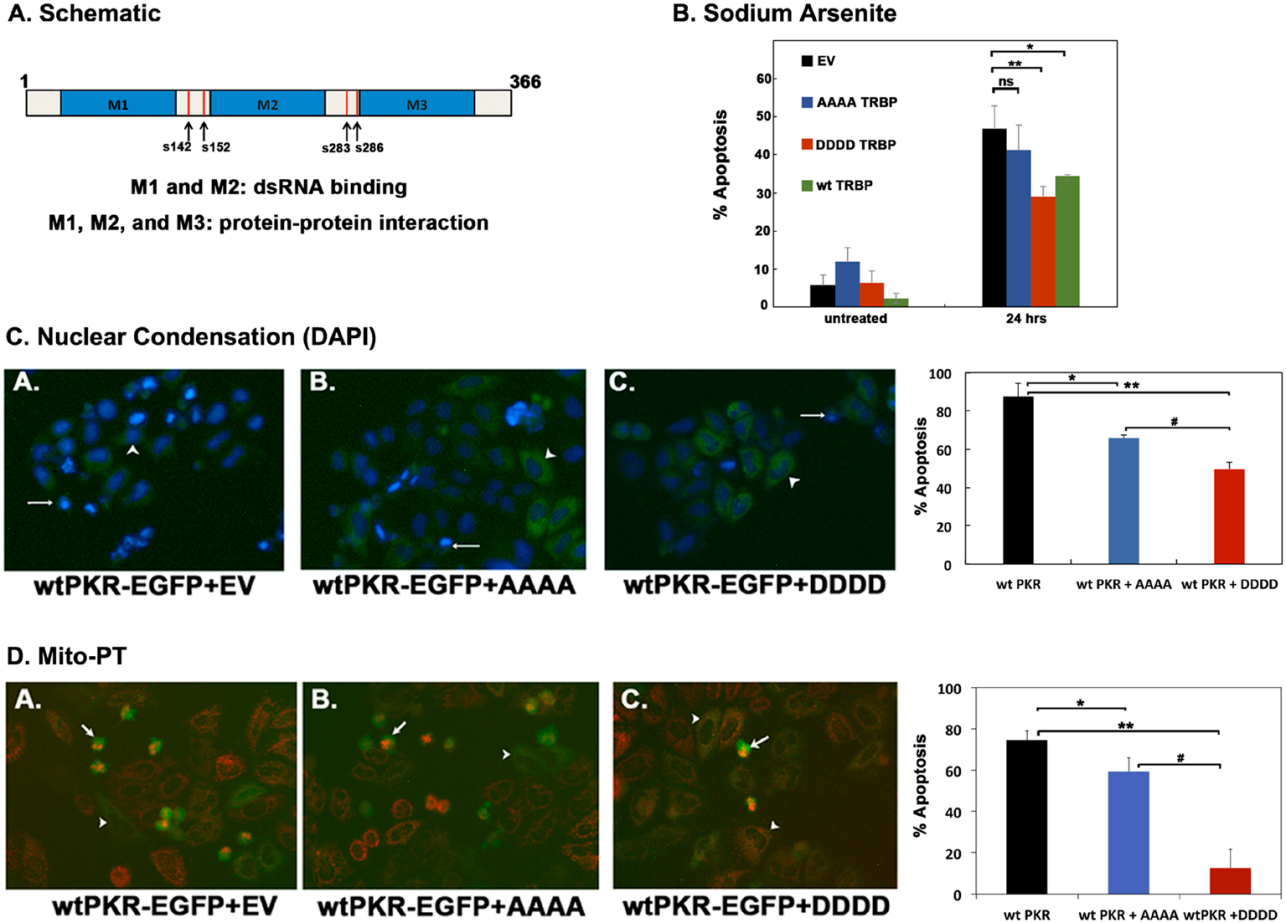
TRBP phosphorylation inhibits PKR-mediated apoptosis during cell stress. (**A**) Schematic representation of TRBP phosphorylation sites. Blue boxes represent the three double-stranded RNA binding motifs (dsRBMs), M1, M2, and M3. Red vertical lines represent previously identified ERK 1/2 phosphorylation sites at S142, S152, S283, and S286. (**B**) Expression of phospho-mimic TRBP protects cells during oxidative stress. HeLa cells were transfected with 200 ng pEGFPC1 (EV) alone (black bars) or with 200 ng each of pEGFPC1 and Flag TRBP AAAA/pcDNA 3.1^−^ (blue bars), 200 ng each of pEGFPC1 and Flag TRBP DDDD/pcDNA 3.1^−^ (red bars) or 200 ng each of pEGFPC1 and Flag wt TRBP/pcDNA 3.1^−^ (green bars). 24 hours after transfection, the cells were treated with 25 μM sodium arsenite, fixed and stained with DAPI nuclear stain. At least 300 EGFP-positive cells were scored as apoptotic or live based on nuclear condensation indicated by intense DAPI nuclear staining and cell morphology. The percentage of cells undergoing apoptosis (% apoptosis) was calculated using the formula: (EGFP- expressing cells with intense DAPI nuclear staining/Total EGFP-expressing cells) x 100. Bars represent averages ± S.D. from three independent experiments. One-way ANOVA followed by post-hoc Tukey test was performed, ns: not significant, asterisk *p value of 0.043. asterisk **p value of 0.007. (**C**) and (**D**) Phospho-mimic TRBP inhibits PKR mediated apoptosis more efficiently than phospho-defective TRBP (**C**) HeLa cells were plated on coverslips and transfected with 500 ng of wt PKR/pEGFPC1 and 20 ng of empty vector pCDNA3.1^−^ (wt PKR; black bar) or with 500 ng of wt PKR/pEGFPC1 + 20 ng of Flag TRBP AAAA/pcDNA 3.1^−^ (blue bar) or 500 ng of wt PKR/pEGFPC1 + 20 ng of Flag TRBP DDDD/ pcDNA 3.1^−^ (red bar). 24 hours after transfection, the cells were fixed and mounted in Vectashield mounting media with DAPI nuclear stain. Representative fluorescent micrographs of HeLa cells transfected with wt PKR pEGFPC1 alone (Panel A), or in combination with Flag TRBP AAAA/pcDNA 3.1^−^ (Panel B) or Flag TRBP DDDD/pcDNA 3.1^−^ (Panel C) are shown. At least 500 EGFP-PKR expressing cells showing green fluorescence were scored as apoptotic (white arrows) or live (white arrowheads) based on nuclear condensation indicated by intense DAPI staining and cellular morphology. The cells showing intense DAPI staining and rounded morphology were scored as apoptotic. The percentage of cells undergoing apoptosis was determined as described in (**A**). Bars represent averages ± S.D. from three independent experiments. One-way ANOVA followed by post-hoc Tukey test was performed, asterisk *p value 0.0034, double asterisk **p value 0.0002, ^#^p value 0.0134. (**D**) Phospho-mimic TRBP expression abrogates mitochondrial depolarization during PKR-mediated apoptosis. HeLa cells were plated on coverslips and transfected with 500 ng of wt PKR/pEGFPC1 and 20 ng of empty vector pCDNA3.1^−^ (wt PKR; black bar) or with 500 ng of wt PKR/pEGFPC1 + 20 ng of Flag TRBP AAAA/pcDNA 3.1^−^ (blue bar) or 500 ng of wt PKR/pEGFPC1 + 20 ng of Flag TRBP DDDD/ pcDNA 3.1^−^ (red bar). 24 hours after transfection, changes in the mitochondrial potential of transfected cells were assessed using the MitoPT TMRM Assay kit and observed by fluorescence microscopy. Representative fluorescent micrographs of the cells transfected with wt-PKR pEGFPC1 alone (wt-PKR-EGFP + EV, Panel A), and AAAA TRBP (wt-PKR-EGFP + AAAA, Panel B) or DDDD TRBP (wt-PKR-EGFP + DDDD, Panel C) are represented. At least 500 PKR expressing cells (GFP positive, green fluorescent cells) were scored as live (white arrowheads) or dead (white arrows) based on decreased or absent red fluorescence. The PKR expressing, green fluorescent cells that showed diminished or no MitoPT staining (red fluorescence) were counted as apoptotic and the green fluorescent cells that showed good MitoPT staining (red fluorescence) were counted as live. The percentage of cells undergoing apoptosis (% apoptosis) was calculated using the formula: (EGFP- expressing cells with decreased or absent red fluorescence/Total EGFP-expressing cells) x 100. Bars represent averages ± S.D. from three independent experiments. One-way ANOVA followed by post-hoc Tukey test was performed, asterisk *p value 0.0108, double asterisk **p value 0.0003, ^#^p value 0.0002.

Having demonstrated that TRBP is phosphorylated by JNK and ERK 1/2 in response to oxidative stress, we wanted to determine how TRBP phosphorylation affects the oxidative stress and PKR-mediated cellular apoptosis. To evaluate the involvement of phosphorylation, we generated a phospho-defective TRBP point mutant (TRBP AAAA) which contains alanine for serine substitution at 4 sites (S142, S152, S283, S286) previously identified as MAPK/ERK 1/2 substrate sites^34^. Of these sites, S142 and S152 have also been previously shown to be phosphorylated by JNK^33^. A phospho-mimic TRBP point mutant (TRBP DDDD) was also generated by substituting aspartic acid for serine at the same four sites (Fig. 3A).

To examine the effect of TRBP phosphorylation on oxidative stress-induced apoptosis, we transfected HeLa cells with each TRBP phospho-mutant as well as wt TRBP and observed changes in the induction of apoptosis in response to oxidative stress. As seen in Fig. 3B, the cells transfected with the empty vector (EV) alone showed 5.6% apoptosis (Untreated, black bar) in the absence of stress which increased to 46.9% at 24 after sodium arsenite treatment (24 hrs, black bar). The DDDD TRBP phospho-mimic mutant showed significantly reduced apoptosis in response to oxidative stress with only 29.1% cells undergoing apoptosis at 24 h after treatment (24 hrs, red bar). Statistical analysis also showed a significant difference between the % apoptosis in the cells expressing the empty vector as compared to the cells expressing the TRBP phospho-mimic mutant, indicating that TRBP phosphorylation does have a protective effect on cells during oxidative stress. Analysis of the % apoptosis in the cells expressing the AAAA TRBP phospho-defective mutant to that of cells expressing EV showed no statistical difference (24 hrs, blue bar), highlighting the importance of phosphorylation for TRBP’s anti-apoptotic activity. The cells overexpressing the wt TRBP showed 34.3% apoptosis, which is more compared to the cells overexpressing DDDD TRBP, but less than the cells overexpressing AAAA TRBP. This suggests that the wt TRBP overexpression allows for higher amounts of phospho-TRBP than the levels with endogenous TRBP in EV transfected cells, thus making it more effective that AAAA TRBP in preventing prolonged phosphorylation of eIF2α and apoptosis.

We next examined the effect of TRBP phosphorylation on PKR-induced apoptosis. An overexpression of active PKR in mammalian cells is sufficient to trigger cellular apoptosis in the absence of any stress signals^39,40^. It has been previously observed by us and others that a PKR-EGFP fusion construct encodes a constitutively active PKR, which induces apoptosis when transfected in mammalian cells^41^. Thus, cells were transfected with a constitutively active PKR expression plasmid (wt-PKR-EGFP+EV) or in combination with the TRBP AAAA phospho-defective or DDDD phospho-mimic mutant and assayed for changes in apoptosis induced by active PKR. We used nuclear condensation as the hallmark sign of apoptosis as indicated by the intense DAPI nuclear fluorescence^42^. There is a significant amount of apoptosis at 87.5% (Fig. 3C, wt PKR, black bar) in transfected cells that express constitutively active PKR. The percentage cell death is significantly reduced with the co-expression of both AAAA phospho-defective (65.9%) and DDDD phospho-mimic TRBP (49.5%) mutants with PKR-EGFP (Fig. 3C, wt PKR + AAAA and wt PKR + DDDD, blue and red bars). Consistent with our previous results in Fig. 3A, we also observe greater reduction in apoptosis with expression of the TRBP phospho-mimic mutant, indicating that although AAAA phospho-defective mutant can still inhibit PKR, the DDDD phosphor-mimic mutant inhibits PKR much more efficiently.

We further assayed apoptosis by using the mitochondrial membrane depolarization as an early marker for apoptotic cells^43^. The effect of TRBP phospho-mutants (TRBP AAAA or TRBP DDDD) on apoptosis induced by constitutively active PKR-EGFP was measured. Similar to our result in Fig. 3C, we observed apoptosis (~75%) in cells expressing constitutively active PKR. Cells co-expressing the AAAA TRBP phospho-defective mutant had a 16% decrease in cell death compared to the cells expressing PKR-EGFP alone, while the cells co-expressing the DDDD TRBP phospho-mimic mutant had a 63% decrease in cell apoptosis compared to the cells expressing PKR-EGFP alone. The DAPI nuclear condensation assay shows lesser difference between AAAA TRBP and DDDD TRBP as this is a late marker of apoptosis, whereas the Mito-PT assay is measuring an early marker of apoptosis, which shows a more pronounced difference between AAAA TRBP and DDDD TRBP. Taken together, these results clearly demonstrate that TRBP phosphorylation is protective during oxidative stress, and this protection is mediated via inhibition of PKR.

### TRBP phosphorylation inhibits PKR’s kinase activity more efficiently

**Figure 4:**
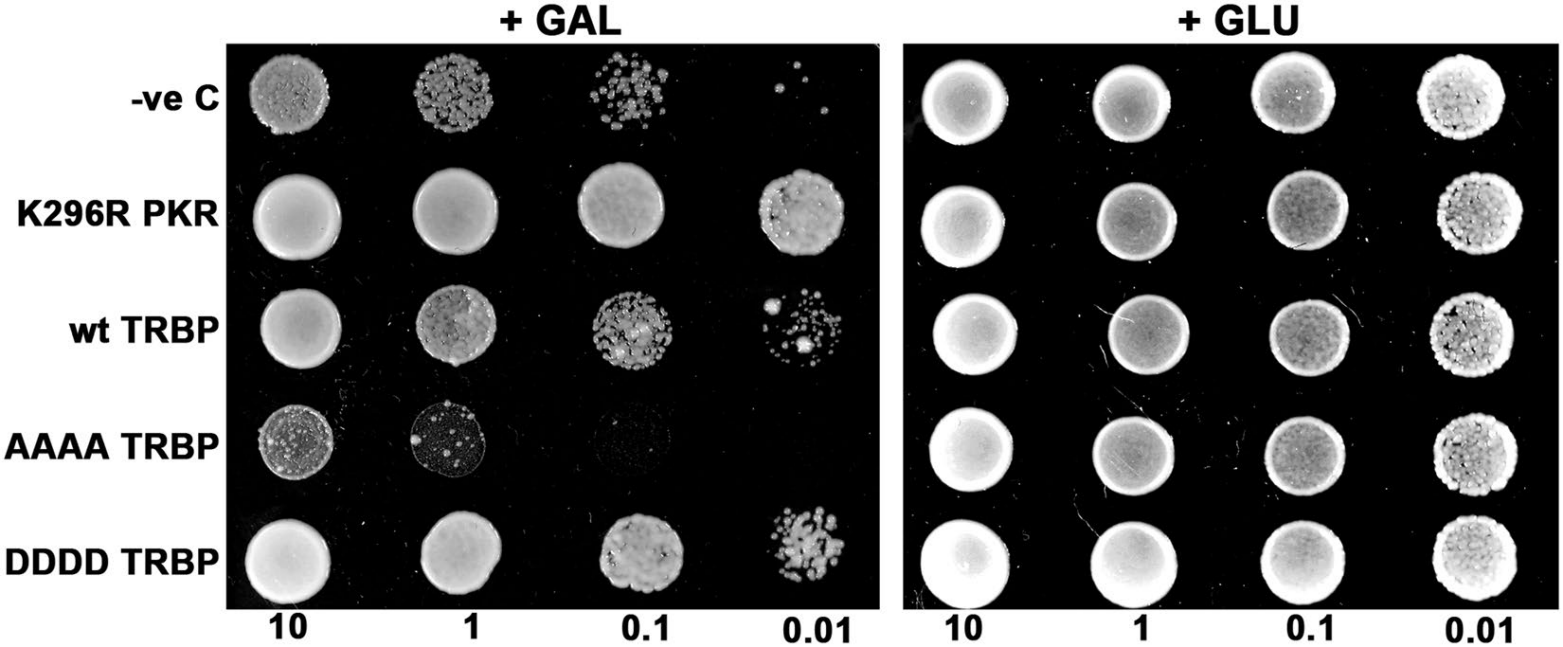
The phosphorylated TRBP isoform efficiently reverses PKR’s growth inhibition phenotype in yeast. (**A**) Yeast growth inhibition assay. Yeast INVSc1 cells were co-transformed with wt PKR/pYES2 and empty vector pYES3CT (PKR alone), wt PKR/pYES2 and K296R PKR/pYES3CT (PKR+K296R), wt PKR/pYES2 and wt TRBP/pYES3CT (PKR+wtTRBP), wt PKR/pYES2 and AAAA TRBP/pYES3CT (PKR+AAAA), or wt PKR/pYES2 and DDDD TRBP/pYES3CT (PKR + DDDD). Ten microliters of transformed yeast cells (OD_600_ = 10, 1, 0.1, 0.01) were spotted on double dropout media (-uracil, - tryptophan) with either glucose (+GLU) or galactose (+GAL) as sole carbon source. Plates were incubated for three days at 30 °C. Transformation of INVSc1 with wt PKR/pYES2 and empty vector pYES3CT served as a control showing growth inhibition on galactose plates, while transformation with wtPKR/pYES2 and K296R PKR/pYES3CT served as a positive control for inhibition of PKR and a reversal of growth inhibition phenotype.

To determine if protection from apoptosis was a direct result of enhanced PKR inhibition by the phosphorylated TRBP isoform, we performed a yeast growth inhibition assay using the INVSc1 *S. cerevisiae* yeast strain. The expression of active PKR in *S. cerevisiae* suppresses yeast growth, and this growth inhibition can be reversed by co-expression of PKR inhibitors such as the dominant negative PKR mutant, K296R^12,23,44^. We introduced a galactose-inducible wt PKR yeast expression plasmid (wt PKR/pYES2) in combination with K296R, wt TRBP, AAAA TRBP, or DDDD TRBP expression plasmids (pYES3CT) into INVSc1 yeast cells. As seen in Fig. 4, induction of PKR expression on galactose-containing media inhibited yeast cell growth (+GAL panel, wt PKR alone). We also observed that co-expression of K296R or wt TRBP reversed the PKR-mediated growth phenotype (+GAL panel, K296R, wt TRBP) in accordance with previous reports that have shown that K296R and TRBP inhibit PKR activity^32,45^. Interestingly, when we co-expressed the phospho-deficient TRBP mutant (AAAA TRBP), it was unable to reverse the growth phenotype (compare wt PKR alone to AAAA TRBP, +GAL panel) suggesting that TRBP phosphorylation is crucial for TRBP’s ability to inhibit PKR. On the other hand, co-expression of the phospho-mimic TRBP mutant (DDDD TRBP) reversed the PKR-mediated growth inhibition more efficiently as compared to wt TRBP. It is interesting that the inhibition of PKR dependent growth suppression by wt TRBP is less efficient than DDDD TRBP but more efficient than AAAA TRBP. This would indicate that the wt TRBP is being phosphorylated in yeast and depending on what percentage of wt TRBP is phosphorylated, it is able to inhibit PKR more efficiently than AAAA TRBP but less efficiently that DDDD TRBP. These results indicate that phosphorylated TRBP inhibits PKR’s kinase activity in a more efficient manner.

### Stress induced TRBP phosphorylation enhances TRBP-PKR interactions, while weakening TRBP-TRBP interactions

**Figure 5:**
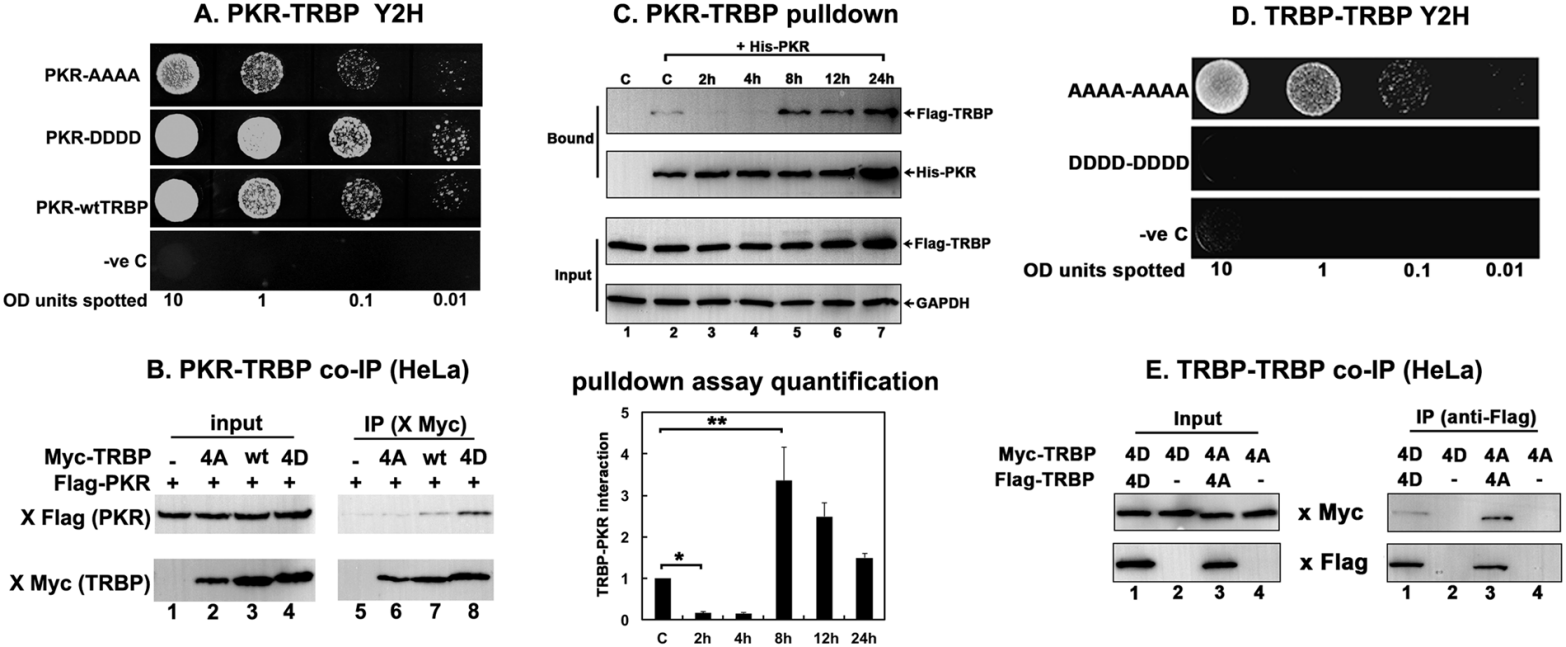
TRBP phosphorylation strengthens PKR-TRBP interaction and weakens TRBP-TRBP interaction. (**A**) Phospho-mimic TRBP mutant interacts stronger with PKR compared to the phospho-defective TRBP mutant in yeast two-hybrid assay. PKR/pGAD424 and either AAAA TRBP/pGBKT7, DDDD TRBP/pGBKT7, or wt TRBP/pGBKT7 were co-transformed into AH109 yeast cells and selected on SD double dropout media (-tryptophan, - leucine). Ten microliters of transformed yeast cells (OD_600_ = 10, 1, 0.1, 0.01) were spotted on SD triple dropout media (-tryptophan, - leucine, - histidine) containing 10 mM 3-amino-1,2,4-triazole (3-AT). Plates were incubated for 3 days at 30 °C. Transformation of PKR in pGAD424 and pGBKT7 empty vector served as a negative control. (**B**) Phosphomimic TRBP mutant shows stronger heteromeric interaction with PKR compared to the phosphodefective TRBP mutant in mammalian cells. HeLa cells were transfected with Flag K296R PKR/pcDNA 3.1^−^ and either myc TRBP AAAA/pcDNA 3.1^−^, myc wt TRBP/pcDNA 3.1^−^, or myc TRBP DDDD/pcDNA 3.1^−^. The cells were harvested 24 hours after transfection, and myc AAAA, DDDD or wt TRBP was immunoprecipitated using anti-myc monoclonal antibody conjugated agarose beads. Co-immunoprecipitated Flag PKR was analyzed by western blot analysis with an anti-Flag antibody (IP: x Flag (PKR) panel). The blot was subsequently re-probed with anti-myc antibody to ensure equal myc TRBP immunoprecipitation from each sample (IP: x myc (TRBP) panel). Equal Flag PKR and myc TRBP expression in all samples was tested by western blot analysis of equal amounts of total cell lysate with anti-myc, and anti-Flag antibodies (input: x Flag (PKR) and x myc (TRBP) panels). (**C**) Changes in TRBP association with PKR. Flag TRBP overexpressing cells were treated with 25 μM sodium arsenite for the indicated time points. Cell extracts were prepared in the presence of a phosphatase inhibitor and 25 μg of cell extract was incubated with 500 ng of pure recombinant hexahistidine (His)-tagged PKR immobilized on Ni^2+^-agarose beads. After washing the beads, PKR-associated Flag TRBP was analyzed by SDS polyacrylamide gel electrophoresis followed by western blot analysis with anti-Flag antibody. Western blot analysis was also performed with anti-His antibody to ensure equal His- PKR in each sample. 25 μg of cell extract was also analyzed by western blot analysis with anti-Flag and anti-GAPDH antibodies to ensure equal addition of cell lysate for each pull down (Input). Quantification of TRBP-PKR pull down: Band intensities were quantified using ImageQuant TL Software, and the ratios of bound TRBP to bound PKR across all samples were calculated and normalized to the band intensities of Flag-TRBP input for each sample. Bound TRBP/his-PKR ratios for all samples were all expressed relative to the control sample (Lane 2). Averages from three independent experiments are plotted as bar graphs ± S.D. One-way ANOVA followed by post-hoc Tukey test was performed, asterisk *p value 0.0000012 and double asterisk **p value 0.0066374. (**D**) Phosphomimic TRBP mutant shows stronger homomeric interaction compared to the phosphodefective TRBP mutant in yeast two-hybrid assay. AAAA TRBP or DDDD TRBP point mutants in pGADT7 and pGBKT7 were co-transformed into AH109 yeast cells and selected on SD double dropout media (-tryptophan, -leucine). Ten microliters of transformed yeast cells (OD_600_ = 10, 1, 0.1, 0.01) were spotted on SD triple dropout media plate (tryptophan, -leucine, -histidine) containing 10 mM 3-amino-1,2,4-triazole (3-AT). Plates were incubated for 5 days at 30 °C. Transformation of pGADT7 and pGBKT7 empty vectors served as a negative control. (**E**) Phosphomimic TRBP mutant shows stronger homomeric interaction compared to the phosphodefective TRBP mutant in mammalian cells. HeLa cells were transfected with either myc TRBP DDDD/pcDNA 3.1^−^ and Flag TRBP DDDD/pcDNA 3.1^−^ or Flag TRBP AAAA/pcDNA 3.1^−^ and myc TRBP AAAA/pcDNA 3.1^−^. The cells were harvested 24 hours after transfection, and Flag TRBP AAAA or DDDD was immunoprecipitated using anti-Flag monoclonal antibody conjugated agarose beads. The co-immunoprecipitation of myc-TRBP was analyzed by western blot analysis with an anti-myc antibody (IP: x Myc panel). Blot was subsequently stripped and re-probed with anti-Flag antibody to ensure equal Flag-TRBP immunoprecipitation from each sample (IP: x Flag panel). Equal AAAA TRBP and DDDD TRBP expression in all samples was tested by western blot analysis of equal amounts of total cell lysate with anti-myc, and anti-Flag antibodies (Input: x Myc and x Flag panels).

To understand how stress-induced phosphorylation of TRBP affects its interaction with PKR, we used the yeast-two hybrid system to test the strength of interaction between PKR and TRBP phospho-mimic and phospho-defective point mutants. The TRBP DDDD and TRBP AAAA point mutants were expressed in yeast as GAL4 DNA binding domain fusion proteins (pGBKT7) with PKR was expressed as a GAL4 activation domain fusion protein. In this system, a stronger interaction between TRBP and PKR is indicated by increased yeast growth in media lacking tryptophan, leucine, and histidine in the presence of the imidazole glycerol-phosphate dehydratase competitive inhibitor, 3-Amino-1,2,4-triazole (3-AT). As seen in Fig. 5A, the TRBP DDDD point mutant shows significantly stronger interaction with PKR as compared to the TRBP AAAA point mutant, suggesting that the stronger PKR inhibition by DDDD TRBP we observed in Figs 3 and 4 results from an enhanced TRBP-PKR interaction. In this assay, the interaction of wt TRBP with PKR is stronger than the interaction between AAAA TRBP and PKR but weaker than the interaction between DDDD TRBP and PKR. These results indicate that the wt TRBP may be phosphorylated somewhat inefficiently by a MAP kinase family member in yeast, resulting in an intermediate level of interaction between PKR and TRBP.

We further confirmed the effect of phospho-deficient or phospho-mimic mutations on TRBP’s interaction with PKR using a co-immunoprecipitation assay from mammalian cell extracts. As seen in Fig. 5B, we observed that significantly more flag-PKR co-immunoprecipitated with myc-DDDD TRBP than with myc-AAAA TRBP or with myc-wt TRBP (IP Flag panel, Lanes 6–8). The Myc panels demonstrate that comparable amounts of Myc-TRBP were immunoprecipitated (IP Myc panel, lanes 6–8), thereby confirming that the significant difference seen in the co-immunoprecipitated PKR bands reflects difference in TRBP-PKR interactions. The absence of co-immunoprecipitated Flag-PKR without co-expression of Flag-TRBP (IP, Lane 5) rules out any nonspecific immunoprecipitation of PKR. Also, similar to the results in Fig. 5A, wt TRBP showed intermediate level of interaction with PKR in mammalian cells, which was stronger than AAAA TRBP but weaker than DDDD TRBP. This indicates that as wt TRBP is capable of getting phosphorylated, its interaction level with PKR will depend on what percentage of total wt TRBP is in phosphorylated state whereas 0% of AAAA TRBP and 100% of DDDD TRBP will behave as phospho-TRBP. Taken together, these results strongly demonstrate that stress-induced TRBP phosphorylation significantly strengthens the interaction between TRBP and PKR, which prevents sustained PKR activation and inhibits excessive apoptosis.

Since we showed enhanced interaction with PKR resulting in more efficient PKR inhibition with the phospho-mimic DDDD TRBP mutant, we tested if we observe significantly increased TRBP-PKR interaction in response to arsenite at 8 and 12 hours post-treatment when we observe the strongest TRBP phosphorylation (Fig. 2A). We tested this by assaying if TRBP from treated cells interacts better with PKR in a pull-down assay. As seen in Fig. 5C, in the absence of stress signals there is weak interaction between PKR and TRBP (Bound panels, Lane 2) which substantially decreases at 2 and 4 hours (Bound panels, Lanes 3 and 4) due to PKR’s disassociation from TRBP in response to stress. We observed much stronger re-association between PKR and TRBP at 8 hours and 12 hours after treatment (Bound panels, Lanes 5 and 6). A quantification of the pull down assay shown as a bar graph shows that both the reduction in TRBP-PKR interaction after stress and the reassociation of TRBP with PKR at later time points after stress is statistically significant. Thus, our results suggest that TRBP’s interaction with PKR and consequently its ability to inhibit PKR effectively during cell stress is closely linked to its phosphorylation status.

Since stress-induced phosphorylation is essential for efficient PACT-PACT interactions^27^, we investigated how phosphorylation affects TRBP-TRBP interactions. We expressed both TRBP phospho-mutants in yeast as GAL4 DNA-binding domain fusion proteins (pGBKT7) and GAL4 activation domain fusion proteins (pGADT7) and assayed for the strength of TRBP AAAA and TRBP DDDD homomeric interactions by the amount of yeast growth on nutrient deficient media in the presence of 3-AT. As seen in Fig. 5D, yeast cells expressing both AAAA TRBP expression vectors show growth at all dilutions, indicating strong homodimer interaction between unphosphorylated TRBP proteins. In contrast to this, there was a complete absence of growth even at the most concentrated dilution of yeast cells (10 OD) expressing both DDDD TRBP yeast expression vectors, which indicates that TRBP phosphorylation is unfavorable to the formation of TRBP homodimers.

We also examined TRBP AAAA and TRBP DDDD homomeric interactions in mammalian cells using a co-immunoprecipitation assay. As seen in Fig. 5E, we observed that significantly less myc-DDDD TRBP was co-immunoprecipitated with Flag-DDDD TRBP (IP Myc panel, Lane 1) compared to the myc-AAAA TRBP co-immunoprecipitated with Flag-AAAA TRBP (IP Myc panel, Lane 3). The Flag panels demonstrate that comparable amounts of Flag-DDDD TRBP (IP panel, Lane 1) and Flag-AAAA TRBP (IP panel, Lane 3) were immunoprecipitated, thereby confirming that the significant difference seen in co-immunoprecipitated bands reflects difference in TRBP homomeric interactions. The absence of co-immunoprecipitated myc-DDDD or myc-AAAA TRBP without co-expression of Flag-DDDD TRBP (IP, Lane 2) or Flag-AAAA TRBP (IP, Lane 4) rules out any nonspecific immunoprecipitation. Taken together, these results demonstrate that stress-induced TRBP phosphorylation weakens homomeric interactions between TRBP molecules while simultaneously enhancing TRBP-PKR interactions, which plays an important role in attenuating sustained PKR activation during cell stress and inhibits excessive apoptosis. The results presented here contribute to our understanding of how PKR activity is regulated negatively at later time points after oxidative stress to prevent excessive apoptosis.

## Discussion

Activation of PKR during cellular stress is regulated by PACT and TRBP, PACT acting positively to activate PKR and TRBP acting negatively to suppress excessive PKR activity^22,24,25^. Initially at early time points after stress, PACT activates PKR to aid inhibition of protein synthesis via phosphorylation of eIF2α^22,25,46^. Our results presented here, demonstrate that TRBP is phosphorylated by ERK 1/2 and JNK in response to oxidative stress at late time points and the phosphorylated TRBP inhibits PKR’s kinase activity more efficiently to protect cells from apoptosis. In addition, the enhanced PKR inhibition and protection from apoptosis by phospho-TRBP is brought about by an increased interaction between phospho-TRBP and PKR as well as decreased phospho-TRBP homomeric interactions. The timely downregulation of PKR activity and eIF2α phosphorylation is achieved in part by induction of GADD34, a regulatory subunit of protein phosphatase 1 (PP1)^47,48^. Our data indicates that TRBP also plays an important role in the downregulation of PKR activity. Thus, PKR activity during cell stress is dictated not only by stress-induced changes in interactions between PACT and PKR, but also by interactions between PKR and TRBP. TRBP interacts with both PACT and PKR and although TRBP phosphorylation enhances its affinity for PKR while reducing the TRBP-TRBP interactions, we observed no effect of TRBP phosphorylation on TRBP-PACT interactions (data not shown). On the contrary, stress-induced PACT phosphorylation reduces the PACT-TRBP interactions while increasing PACT-PACT interactions and PACT-PKR interactions, thereby leading to PKR activation^25,46^. Strikingly, although TRBP and PACT are very homologous, the stress-induced phosphorylation affects the protein-protein interaction properties of PACT and TRBP quite differently.

**Figure 6:**
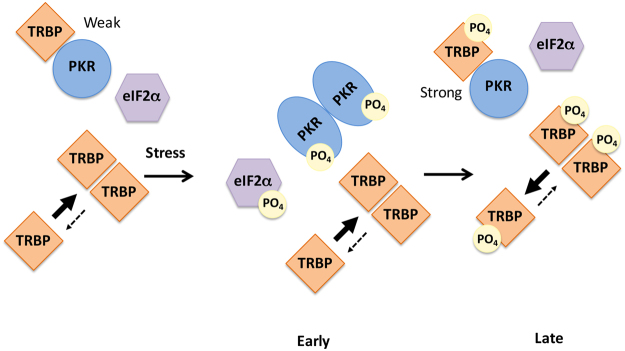
A schematic model of PKR-TRBP interaction in response to oxidative stress. As previously established (refs^19,21,22^), in the absence of stress, TRBP heterodimerizes with PKR, PKR is catalytically inactive and eIF2α is not phosphorylated. At early time points after ER stress, TRBP dissociates from PKR and PKR is activated leading to its autophosphorylation and eIF2α phosphorylation. At late time points after stress, TRBP is phosphorylated by ERK and JNK and interacts with PKR with higher affinity. The cells recover by forming TRBP-PKR heterodimers and turning off PKR and eIF2α phosphorylation. Phosphorylated TRBP largely remains monomeric as TRBP-TRBP interactions are weakened by phosphorylation of TRBP.

Based on our data, we present a schematic model for TRBP-mediated downregulation of PKR activity at late time points after cellular stress. As depicted in Fig. 6, PKR and unphophorylated TRBP interact under unstressed conditions in the cell, preventing PKR activation, and eIF2α phosphorylation. Unphosphorylated TRBP also forms homodimers efficiently. In response to oxidative stress, PKR is activated by phosphorylated PACT homodimers (not depicted) and eIF2α is phosphorylated to bring about a transient protein synthesis inhibition. TRBP at this point remains unphosphorylated, and efficiently forms TRBP-TRBP homodimers. Late after the onset of the stressful event, ERK1/2 and JNK phosphorylate TRBP, leading to significantly decreased TRBP-TRBP interactions, and increased TRBP-PKR interactions. Phospho-TRBP interacts with PKR at significantly higher affinity to bring about efficient PKR inactivation, and eIF2α is dephosphorylated. Phospho-TRBP does not form homodimers efficiently and this could partly explain efficient PKR inhibition as it is established that PKR is activated mainly by trans-autophosphorylation and PKR-PKR interactions. Thus, monomeric phospho-TRBP could potentially function to inhibit PKR by preventing PKR-PKR interactions.

The dsRBM motifs present in PKR, TRBP, and PACT possess the characteristic alpha-beta-beta-beta-alpha fold that has two well-characterized functions to bind structured RNA molecules and to mediate protein-protein interactions^19,49^. This motif is widely distributed in eukaryotic proteins, as well as in proteins from bacteria and viruses and the dsRBM-containing proteins are involved in a variety of cellular processes ranging from RNA editing, nucleocytoplasmic transport, RNA localization, protein phosphorylation in translational control, and contain a variable number of dsRBM domains^50^. In addition, dsRBMs can also recognize non-RNA targets (proteins and DNA), and act in combination with other dsRBMs and non-dsRBM motifs to play a regulatory role in catalytic processes^51^. Our work presented here add one more layer of complexity to the versatility of the dsRBM as it demonstrates that phosphorylation sites residing outside the dsRBM influence the strength of protein-protein interactions mediated via the dsRBMs. This also underscores the importance of optimal juxtaposition of the multiple dsRBM motifs relative to each other in regulating protein interactions as the phosphorylation of specific serines outside the motif can possibly bring about significant changes in overall protein conformations. In other members of the diverse family of dsRBM-containing proteins, the role of phosphorylation and post-translational modifications in regulating interactions with RNA or proteins remains to be investigated in future.

Our results on the effect of TRBP phosphorylation on PKR activity are in agreement with Kim *et al*. who reported that phospho-TRBP efficiently inhibits PKR during M-G1 transition to regulate cell cycle^52^. In this study, the phosphorylation of TRBP during M-G1 transition was shown to be mediated by JNK. Of the four sites we studied, S142 and S152 were also identified by Kim *et al*. to be phosphorylated by JNK during M phase. ERK mediated phosphorylation of TRBP on S142, S152, S283, and S286 was reported in response to mitogenic signaling and was accompanied by a coordinated increase in the levels of growth-promoting miRNAs and a reduction in the levels of tumor suppressor let-7 miRNA^34^. TRBP phosphorylation was has been reported to occur in response to metabolic stress and inhibition of TRBP phosphorylation during metabolic stress reduced inflammation and improved systemic insulin resistance and glucose metabolism^53,54^. However, the exact functions of TRBP and PKR in high-fat diet-induced obesity and associated metabolic and inflammatory complications remains unclear and controversial^55^. TRBP plays an important pro-viral function in HIV infected cells by regulating PKR activity and promoting HIV replication^56,57^. Any effect of TRBP phosphorylation in HIV-infected cells also remains unexplored at present. Our work on the impact of TRBP phosphorylation on the stress signaling pathway and cellular survival thus presents an additional paradigm for exploring the existence and importance of such TRBP-mediated regulatory mechanisms in virus infected cells as well as miRNA expression and function in response to cellular stress.

## Methods

### Reagents, Cell Lines and Antibodies

HeLaM and HeLa Tet off cells were cultured in Dulbecco’s Modified Eagle’s Medium (DMEM) containing 10% fetal bovine serum and penicillin/streptomycin. Transfections were performed with Effectene Transfection Reagent (Qiagen) according to the manufacturer’s protocol.

Tetracycline inducible cell lines stably expressing Flag TRBP were generated by transfection of HeLa Tet off cells with 500 ng of Flag TRBP/pTRE2pur expression plasmid. Selection of puromycin-resistant colonies was carried out 24 hours after transfection by the addition of 700 ng/ml puromycin. Another cell line was also established using the pTRE2 puro plasmid as a control. Doxycycline inducibility was quantified in Flag TRBP/pTRE2pur cell clones after removal of doxycycline by western blot analysis.

Sodium arsenite, phosphatase inhibitor cocktail (Phosphatase Inhibitor Cocktail 2 – P5726), and the JNK inhibitor (SP600125, Catalog number S5567) were purchased from Sigma Aldrich. MEK1/MEK2 inhibitor (PD0325901) was purchased from Calbiochem (444968).

Antibodies used are as follows: mouse monoclonal anti-FLAG M2 HRP (Sigma-Aldrich A8592), mouse monoclonal anti-c-Myc HRP 9E10 (Santa Cruz Biotechnology SC-40), mouse monoclonal anti-polyhistidine clone His-1 HRP (Sigma-Aldrich A7058), mouse monoclonal anti-PKR (R&D systems MAB1980), rabbit polyclonal anti-phospho PKR Thr 451 (Cell Signaling Technology 3075), rabbit monoclonal anti-Phospho-p44/42 MAPK (Erk1/2) (Thr202/Tyr204) (D13.14.4E) XP® (Cell Signaling Technology 4370), rabbit monoclonal anti-p44/42 MAPK (Erk1/2) (137F5) (Cell Signaling Technology 4695), rabbit polyclonal anti-PARP (Cell Signaling Technology 9542), mouse monoclonal anti-β-Actin HRP (Sigma-Aldrich A3854), mouse monoclonal anti-GAPDH HRP (Sigma-Aldrich G9295), goat anti-mouse IgG HRP (Sigma-Aldrich A3682), and goat anti-rabbit IgG HRP (BioRad 170-6515).

### Plasmids

The Flag TRBP/BSIIKS^+^, wt TRBP/pGBKT7, myc wt TRBP/pcDNA 3.1^−^, Flag K296R PKR/pcDNA 3.1^−^, K296R PKR/pGAD424 and PKR /pYES2 expression plasmids were prepared as described previously^25,58,59^. Full length TRBP1 ORF with an N-terminal Flag tag from Flag TRBP/BSIIKS^+^ was inserted into the NotI and EcoRV restriction sites of the tetracycline-responsive vector, pTRE2pur (Clontech) to generate Flag TRBP/pTRE2pur. The phospho-defective (TRBP AAAA) and phospho-mimic (TRBP DDDD) point mutants were generated at S121, S131, S262, and S265 by substituting each serine with alanine or aspartic acid using the following primers:

TRBP sense:

5′-GCTCTAGACATATGGAAATGCTGGCCGCCAACC-3′

S121D antisense:

5′-GTTCCATGGCGGGGTCCCTGGTTAGGACTACAGATGGAACTGGGG-3′

S121A antisense:

5′-GTTCCATGGCGGGGGCCCTGGTTAGGACTACAGATGGAACTGGGG-3′

S131D sense:

5′-CGCCATGGAACTGCAGCCCCCTGTCGACCCTCAGC-3′

S262D S265D antisense:

5′CGGAGCTCACTGAGGACACGGCAGCAGGCAGGGCCCAGGGCACCCAGGTCGCCCAGGTCGCAACTGC-3′

S131A sense:

5′-CGCCATGGAACTGCAGCCCCCTGTCGCCCCTCAGC-3′

S262A S265A antisense:

5′CGGAGCTCACTGAGGACACGGCAGCAGGCAGGGCCCAGGGCACCCAGGGCGCCCAGGGCGCAACTGC-3′

The PCR products were sub-cloned into the pGEMT-Easy vector (Promega) and each sequence was verified. Full length AAAA TRBP and DDDD TRBP point mutants were generated in the pGBKT7 yeast expression vector (Clontech) by three-piece ligation of NdeI-NcoI restriction fragment from S121A or S121D/pGEMT-Easy, NcoI-SacI restriction fragment from S131D S262D S265D or S131A S262A S265A/pGEMT Easy and NdeI-SacI cut TRBP/pGBKT7. Each point mutant was subsequently introduced into the pGADT7 yeast expression vector (Clontech) by insertion of the NdeI-BamHI restriction fragment from AAAA TRBP or DDDD TRBP/pGBKT7 into the NdeI-BamHI restriction sites in pGADT7.

Flag-tagged full length AAAA and DDDD TRBP point mutants in pcDNA 3.1^−^ (Invitrogen) were generated by first introducing the NdeI-BamHI restriction piece from AAAA TRBP/pGBKT7 or DDDD TRBP/pGBKT7 into NdeI-BamHI cut Flag/TRBP BSIIKS^+^, and then inserting the XbaI-BamHI restriction fragment from Flag/AAAA TRBP BSIIKS^+^ or Flag/DDDD TRBP BSIIKS^+^ into the XbaI-BamHI sites in pcDNA 3.1^−^. Myc-tagged full length AAAA and DDDD TRBP point mutants were generated by introducing HincII-BamHI restriction fragments from AAAA TRBP/pGBKT7 and DDDD TRBP/pGBKT7 into the EcoRV-BamHI restriction sites in pcDNA 3.1^−^.

### DNA Fragmentation analysis

5 × 10^6^ Flag TRBP/pTRE2pur and pTRE2pur HeLa tet off cells described in “Reagents, Cell Lines and Antibodies” were treated with 10 μM sodium arsenite for the indicated time points. Cells were collected and washed with ice cold 1 × PBS, and lysed in 100 μl of lysis buffer (10 mM Tris-HCl pH 7.5, 10 mM EDTA, and 0.5% Triton-X 100) for 5 minutes on ice. Lysates were centrifuged at 13200 rpm for 5 minutes, and were incubated with 100 μg Proteinase K at 37 °C for 2 hours. 5 μl of 6 M NaCl and 110 μl of isopropanol were subsequently added to the lysates which were then incubated at −20 °C overnight. The precipitated DNA was then collected by centrifugation at 14,000 rpm for 5 minutes. After the isopropanol was removed from each sample, the DNA was dissolved in 20 μl TE Buffer (10 mM Tris-HCl pH 7.5, 10 mM EDTA). The DNA was incubated with 20 μg/ml RNase A at 37 °C for 1 hour before analysis on a 1.5% agarose gel.

### Western blot analysis

Cells were treated with sodium arsenite alone or in combination with 10 μM MEK1/MEK2 inhibitor PD0325901 (Calbiochem) or 10 μM JNK inhibitor SP600125 and harvested at indicated time points. Cells were washed twice with ice cold 1 × PBS. Harvested cells were lysed in western lysis buffer (2% Triton X-100, 20 mM Tris–HCl pH 7.5, 100 mM KCl, 200 mM NaCl, 4 mM MgCl_2_, 40% Glycerol, and phosphatase inhibitor cocktail 2 (Sigma) at 1:100 dilution) for 5 minutes on ice. Lysates were centrifuged at 13,200 rpm for 2 minutes. Protein concentration in the supernatant was quantified using Bradford reagent. Western blot was performed with the indicated antibodies and western blot images were analyzed using the Typhoon FLA 7000 and ImageQuant LAS 4000 (GE Health).

### TRBP-PKR pull-down assay

Flag TRBP/pTRE2pur HeLa Tet off cells grown to 50% confluency in 100-mm dishes were treated with 25 μM sodium arsenite for the indicated time points. Cell extracts were prepared in 100 μl co-immunoprecipitation buffer (20 mM Tris-HCl pH 7.5, 150 mM NaCl, 1 mM EDTA, 1 mM dithiothreitol (DTT), 1% Triton-X 100, 2% Glycerol, and phosphatase inhibitor cocktail 2 (Sigma) at 1:100 dilution). 25 μg of cell extract was bound to 500 μg of recombinant, hexahistidine-tagged PKR (His-PKR) protein immobilized on Ni^2+^-agarose resin (Novagen) in 100 μl co-immunoprecipitation buffer at 4 °C for 1 hour. The beads were washed in 500 μl of co-immunoprecipitation buffer three times and bound Flag-TRBP was analyzed by western blot analysis using anti-Flag antibody. Blot was then stripped and re-probed with anti-His antibody to ascertain equal His-PKR pull down. 25 μg aliquots of whole cell-lysate were analyzed by western blot analysis with anti-Flag and anti-GAPDH antibodies to ensure that equal amounts of cell lysate were used for immunoprecipitation.

### TRBP-PKR Co-immunoprecipitation assay

HeLa cells were transfected in 6-well culture dishes with (i) 250 ng Flag K296R PKR/pcDNA 3.1^−^ (ii) 100 ng myc AAAA TRBP/pcDNA 3.1^−^ and 250 ng Flag K296R PKR/pcDNA 3.1^−^ (iii) 100 ng myc wt TRBP/pcDNA 3.1^−^ and 250 ng Flag K296R PKR/ pcDNA 3.1^−^ (iv) 100 ng myc DDDD TRBP/pcDNA 3.1^−^ and 250 ng Flag K296R PKR/pcDNA 3.1^−^ using the Effectene reagent (Qiagen). 24 hours after transfection, cell extracts were prepared in co-IP buffer (150 mM NaCl, 20 mM Tris-HCl pH 7.5, 1 mM EDTA, 1% Triton X-100, 20% Glycerol). Myc AAAA TRBP, wt TRBP, and DDDD TRBP were immunoprecipitated with anti-c-myc agarose beads (Santa Cruz Biotechnology) in co- IP buffer overnight on a rotating wheel at 4 °C. The agarose beads were washed 5 times in co-IP buffer and the bound proteins were analyzed by western blot analysis with the anti-c-myc (Santa Cruz Biotechnology) and anti-Flag (Sigma) antibodies.

### TRBP-TRBP Co-immunoprecipitation assay

HeLa cells were co-transfected in 6-well culture dishes with 250 ng each of (i) myc TRBP DDDD/pcDNA 3.1^−^ and Flag TRBP DDDD/pcDNA 3.1^−^, (ii) myc TRBP DDDD/pcDNA 3.1^−^ and pcDNA 3.1^−^ (iii) myc TRBP AAAA/pcDNA 3.1^−^ and Flag TRBP AAAA/pcDNA 3.1^−^, (iv) myc TRBP AAAA/pcDNA 3.1^−^ and pcDNA 3.1^−^ using the Effectene reagent (Qiagen). 24 hours after transfection, cell extracts were prepared in co-IP buffer (150 mM NaCl, 30 mM Tris-HCl pH 7.5, 1 mM MgCl_2_, 10% Glycerol, 0.4% Igepal). Flag TRBP AAAA and Flag TRBP DDDD were immunoprecipitated with anti-Flag mab-agarose (Sigma) in co- IP buffer. The agarose beads were washed 5 times in co-IP buffer. The bound proteins were then analyzed by western blot analysis with the anti-c-myc (Santa Cruz Biotechnology) and anti-Flag (Sigma) antibodies.

### Yeast growth inhibition assay

Wild-type and TRBP phospho-mimic and phospho-defective point mutants were subcloned into the pYES3CT yeast expression plasmid (Invitrogen). Wild-type PKR was subcloned into the pYES2 yeast expression vector (Invitrogen) as previously described for galactose inducible PKR expression. The constructs were introduced into InvSc1 yeast cells (Invitrogen) using the Clontech Yeast Transformation Kit. Transformed yeast cells were grown to an OD_600_ of 2 in YPD media (yeast extract, peptone, and dextrose). 500 μl of each culture was pelleted and resuspended in an appropriate amount of distilled water to yield an OD_600_ of 10. Serial dilutions were then made to yield OD_600_ values of 1, 0.1, and 0.01. 10 μl of each dilution was then spotted onto synthetic medium lacking uracil and tryptophan and containing either glucose or galactose as a carbon source (Clontech).

### Yeast Two-Hybrid Interaction Assay

To test TRBP-PKR interaction, full length K296R PKR was expressed as a GAL4 DNA-activation domain fusion protein from the pGAD424 vector and wt TRBP and the AAAA and DDDD TRBP point mutants were expressed as GAL4 DNA-binding domain fusion proteins from the pGBKT7 vector. Full length AAAA and DDDD TRBP point mutants were expressed as GAL4 DNA-activation domain fusion proteins from the pGADT7 vector and GAL4 DNA-binding domain fusion proteins from the pGBKT7 vector to test TRBP-TRBP interaction. The AAAA TRBP and DDDD TRBP pGBKT7/pGADT7 construct pairs and PKR/pGAD424 and AAAA (DDDD) TRBP/pGBKT7 construct pairs were co-transformed into AH109 yeast cells (Clontech) and the transformed yeast cells were plated on double dropout SD minimal medium lacking tryptophan and leucine. In order to check for the transformants’ ability to grow on triple dropout media, transformed yeast cells were grown to an OD_600_ of 2 in YPD media (yeast extract, peptone, and dextrose). 500 μl of each culture was pelleted and resuspended in an appropriate amount of distilled water to yield an OD_600_ of 10. Serial dilutions were then made to yield OD_600_ values of 1, 0.1, and 0.01. 10 μl of each dilution was then spotted onto triple dropout SD minimal media lacking histidine, tryptophan, and leucine in the presence of 10 mM 3-amino-1,2,4-triazole (3-AT). Plates were incubated at 30 °C for 3 days for the PKR-TRBP interaction assay and 5 days for the TRBP-TRBP interaction assay.

### Apoptosis Assay

HeLa cells were grown to 50% confluency in six-well plates and co-transfected with 200 ng of Flag wt TRBP, TRBP AAAA or TRBP DDDD/pcDNA 3.1^−^ and 200 ng of pEGFPC1 (Clontech) using Effectene (Qiagen). Cells were also co-transfected with 200 ng BSIIKS^+^ (Agilent) and 200 ng pEGFPC1 as a control. The cells were observed for GFP fluorescence 24 hours after transfection using an inverted fluorescence microscope (EVOS® FL Imaging System). Cells were treated with 25 μM sodium arsenite, and cellular morphology was monitored at 1 hour intervals. 12 hours after treatment, the cells were rinsed with ice-cold phosphate buffered saline (PBS) and fixed in 2% paraformaldehyde for 10 minutes. Cells were washed twice in ice-cold PBS and permeabilized with 0.1% Triton-X for 10 minutes, after which the cells were washed twice in ice-cold PBS. Cells were stained with the DAPI nuclear stain (4,6-diamidino-2-phenylindole) at 0.5 μg/ml in PBS for 10 minutes at room temperature in the dark. The cells were rinsed once with PBS and viewed under the fluorescent microscope. At least 300 GFP-positive cells were counted as apoptotic or live based on their morphology. Cells showing normal flat morphology were scored as live, while cells showing cell shrinkage, membrane blebbing, rounded morphology and nuclear condensation with intense fluorescence as apoptotic. The percentage of cells undergoing apoptosis (*Percent apoptosis*) was calculated using the formula: (EGFP- expressing cells with intense DAPI nuclear staining/Total EGFP-expressing cells) × 100.

### PKR-inhibition and apoptosis assay

HeLa cells were grown on coverslips and transfected with 500 ng of wt PKR pEGFPC1 and 20 ng pcDNA 3.1^−^, Flag TRBP AAAA/pcDNA 3.1^−^, or Flag TRBP DDDD/pcDNA 3.1^−^ using Effectene (Qiagen). 24 hours after transfection, the cells were rinsed with ice-cold phosphate buffered saline (PBS) and fixed in 2% paraformaldehyde for 10 minutes. Cells were washed twice in ice-cold PBS and permeabilized with 0.1% Triton-X for 10 minutes, after which the cells were washed twice in ice-cold PBS. The cover slips were mounted in Vectashield mounting medium containing DAPI (Vector Laboratories). Cells were then viewed under the fluorescence microscope (EVOS® FL Imaging System). At least 500 EGFP-positive cells were scored as live or apoptotic as described in ‘Apoptosis Assay’.

### Estimation of Mitochondrial Membrane Potential

HeLa cells were grown to 50% confluency in six-well plates and transfected with 500 ng of wt PKR pEGFPC1 and 20 ng pcDNA 3.1^−^, Flag TRBP AAAA/ pcDNA 3.1^−^, or Flag TRBP DDDD/pcDNA 3.1^−^. MitoPT® TMRM assay was performed using the manufacturer’s instructions (ImmunoChemistry Technologies MitoPT® TMRM Assay Kit). Green fluorescence (EGFP-PKR) and changes in red fluorescence (changes in mitochondrial polarization) were observed under an inverted fluorescence microscope (EVOS® FL Imaging System). At least 500 PKR expressing cells (GFP positive cells) were scored as live or dead based on decreased or absent red fluorescence. The percentage of cells undergoing apoptosis (*Percent apoptosis*) was calculated using the formula: (EGFP- expressing cells with decreased or absent red fluorescence/Total EGFP-expressing cells) × 100.

### Statistical analysis

Statistical significance of western blot quantifications and percent apoptosis were determined by two-tailed Student’s T-test assuming equal variance or one-way ANOVA followed by post-Hoc Tukey test respectively. Figure legends indicate the statistical test used, and p values are denoted by brackets and special characters. Alpha level was p = 0.05.

### Data availability statement

All data generated during this study are included in this published article (and its Supplementary Information files).

## Electronic supplementary material

Supplementary material
