# Supplementary material for: Stress-induced TRBP phosphorylation enhances its interaction with PKR to regulate cellular survival
Source: Sci Rep. 2018 Jan 18;8:1020. doi: 10.1038/s41598-018-19360-8 (PMC5773696; doi:10.1038/s41598-018-19360-8)
Supplement: Supplementary file 1 — Supplementary material [file 41598_2018_19360_MOESM1_ESM.pdf]

Stress-induced TRBP phosphorylation enhances its interaction with PKR to regulate cellular survival

Evelyn Chukwurah, and Rekha C. Patel*
Department of Biological Sciences, University of South Carolina, Columbia, SC, 29208, USA

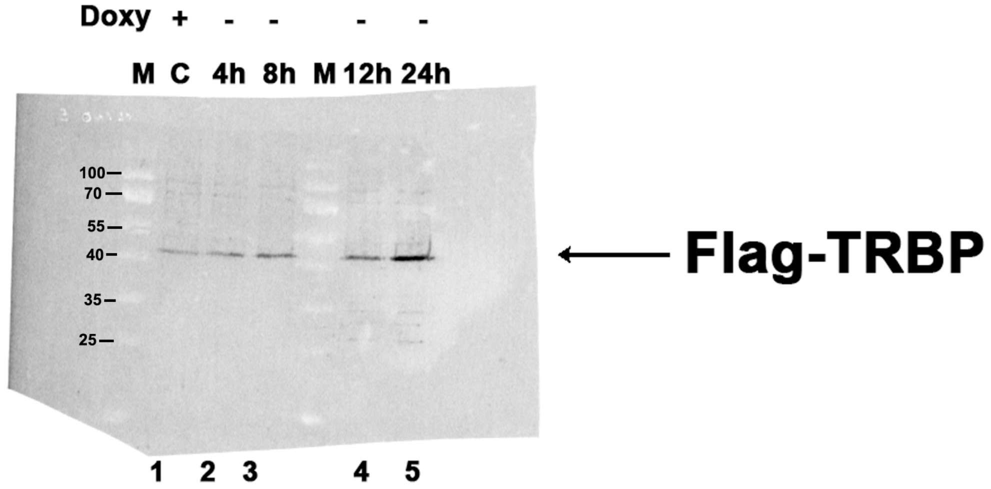

Flag-TRBP

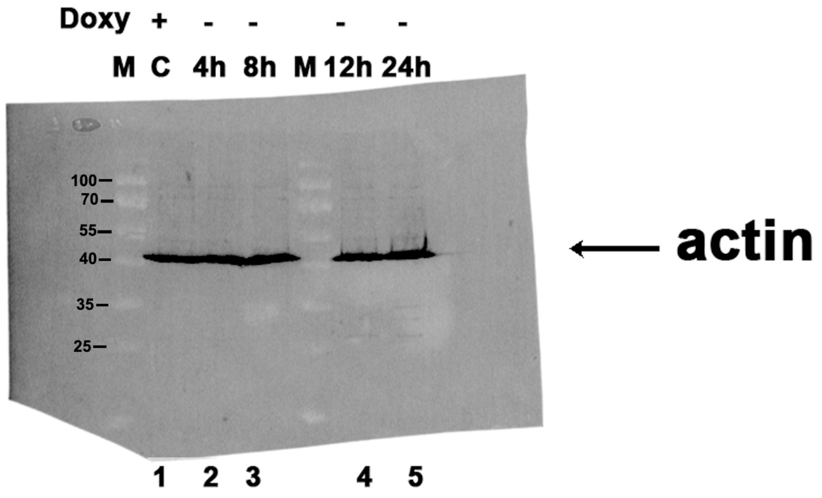

actin

Fig. 1 a

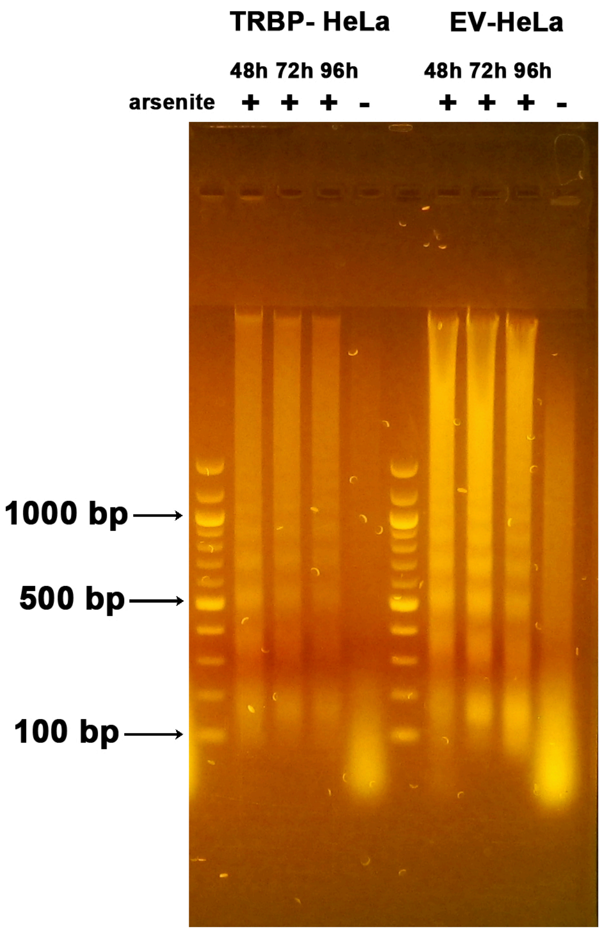

TRBP- HeLa
EV-HeLa
48h 72h 96h
48h 72h 96h
arsenite + + + - + + + -
1000 bp
500 bp
100 bp

Figure 1B

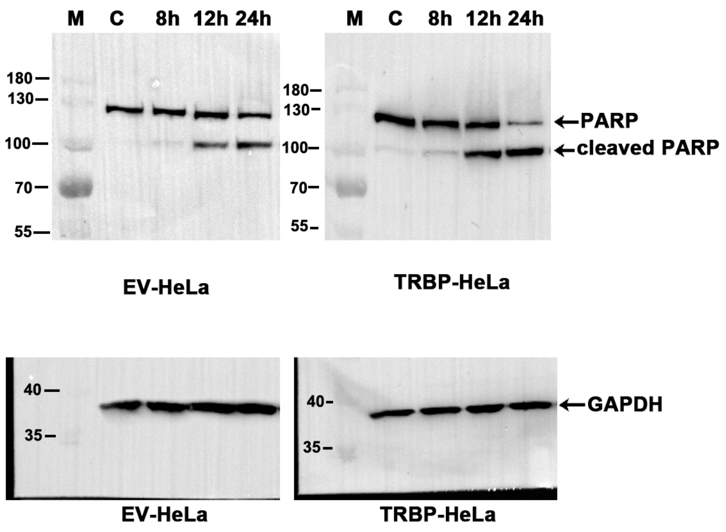

M C 8h 12h 24h
180
130
100
70
55
EV-HeLa
M C 8h 12h 24h
180
130
100
70
55
←PARP
←cleaved PARP
TRBP-HeLa
40
35
EV-HeLa
40
35
←GAPDH
TRBP-HeLa

Figure 1 C

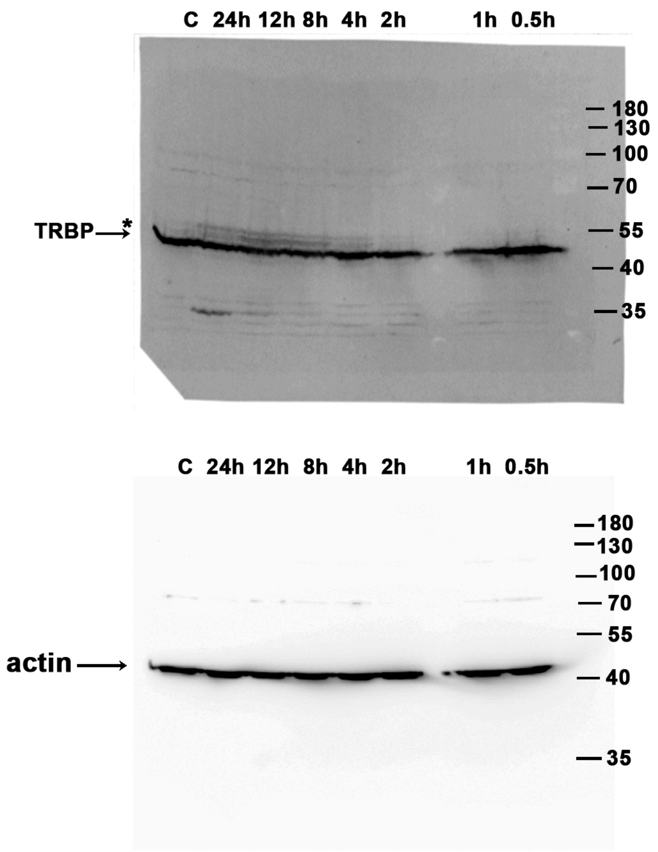
C 24h 12h 8h 4h 2h 1h 0.5h
180
130
100
70
55
40
35
TRBP
*
C 24h 12h 8h 4h 2h 1h 0.5h
180
130
100
70
55
40
35
actin

Figure 2a

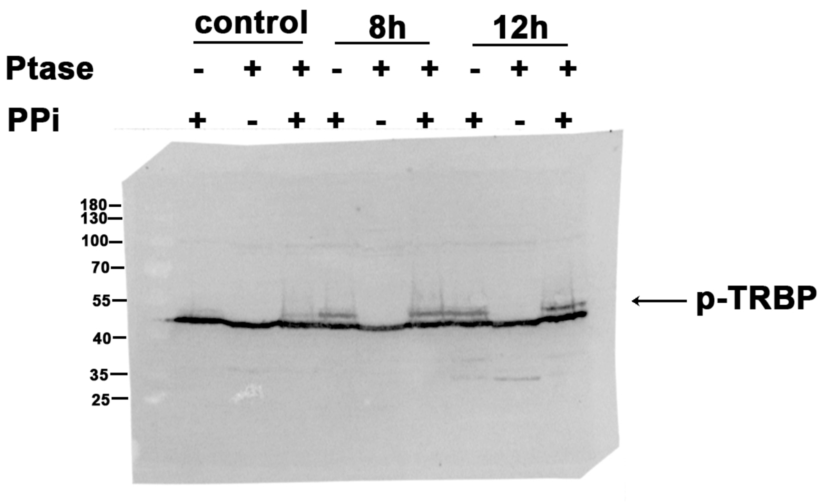

control
8h
12h
Ptase - + + - + + - + +
PPi + - + + - + + - +
180
130
100
70
55
40
35
25
p-TRBP

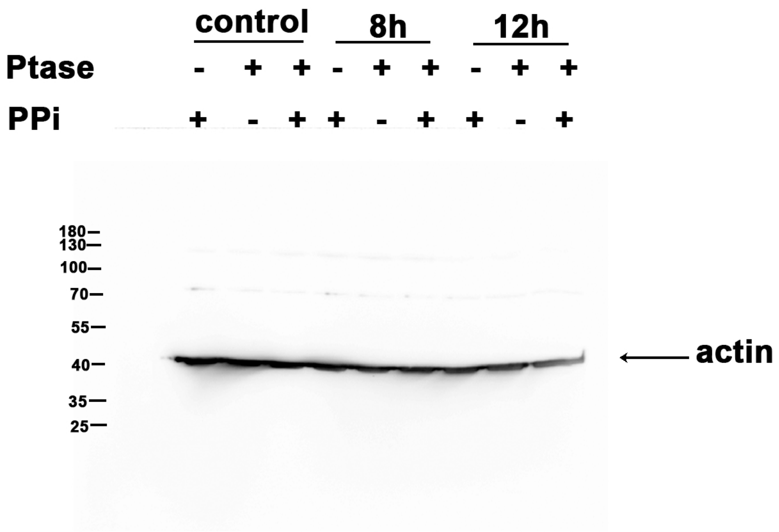

control
8h
12h
Ptase - + + - + + - + +
PPi + - + + - + + - +
180
130
100
70
55
40
35
25
actin

Figure 2c

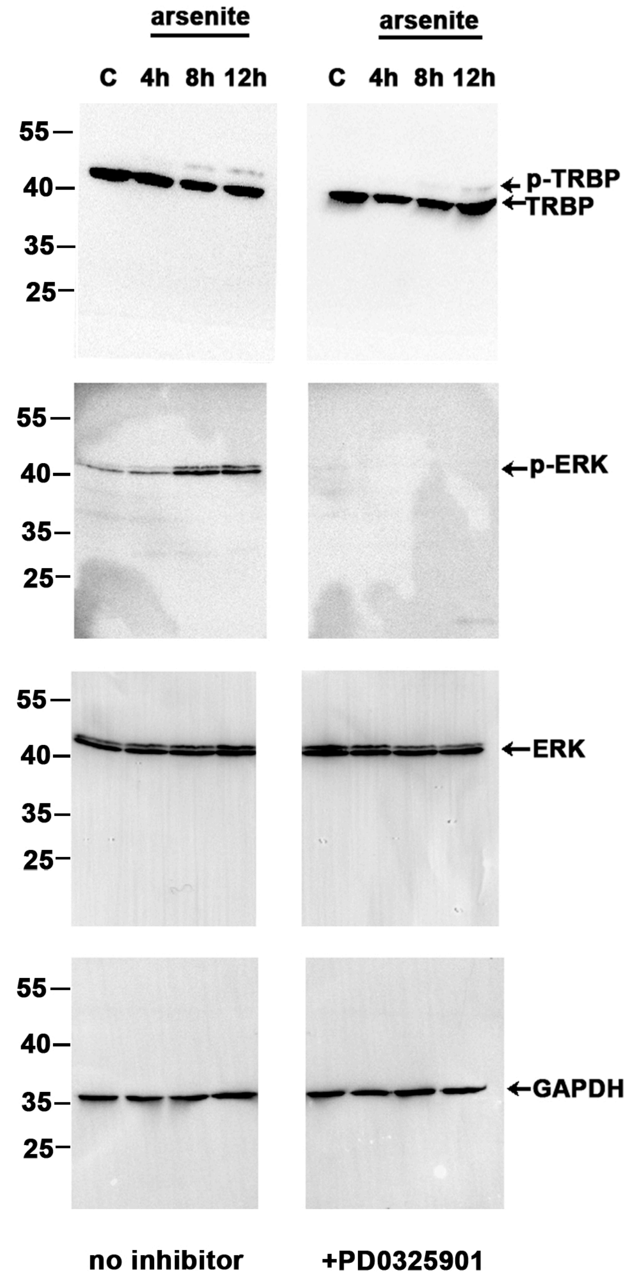
arsenite
C
4h
8h
12h
arsenite
C
4h
8h
12h
55
40
35
25
p-TRBP
TRBP
p-ERK
ERK
GAPDH
no inhibitor
+PD0325901

Figure 2d

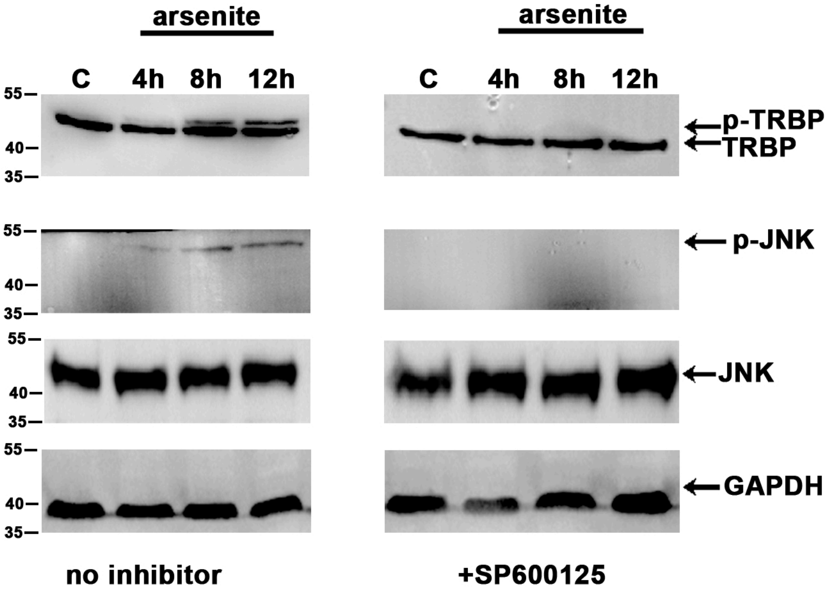
arsenite
arsenite
C
4h
8h
12h
C
4h
8h
12h
55
40
35
p-TRBP
TRBP
p-JNK
JNK
GAPDH
no inhibitor
+SP600125

Figure 2e

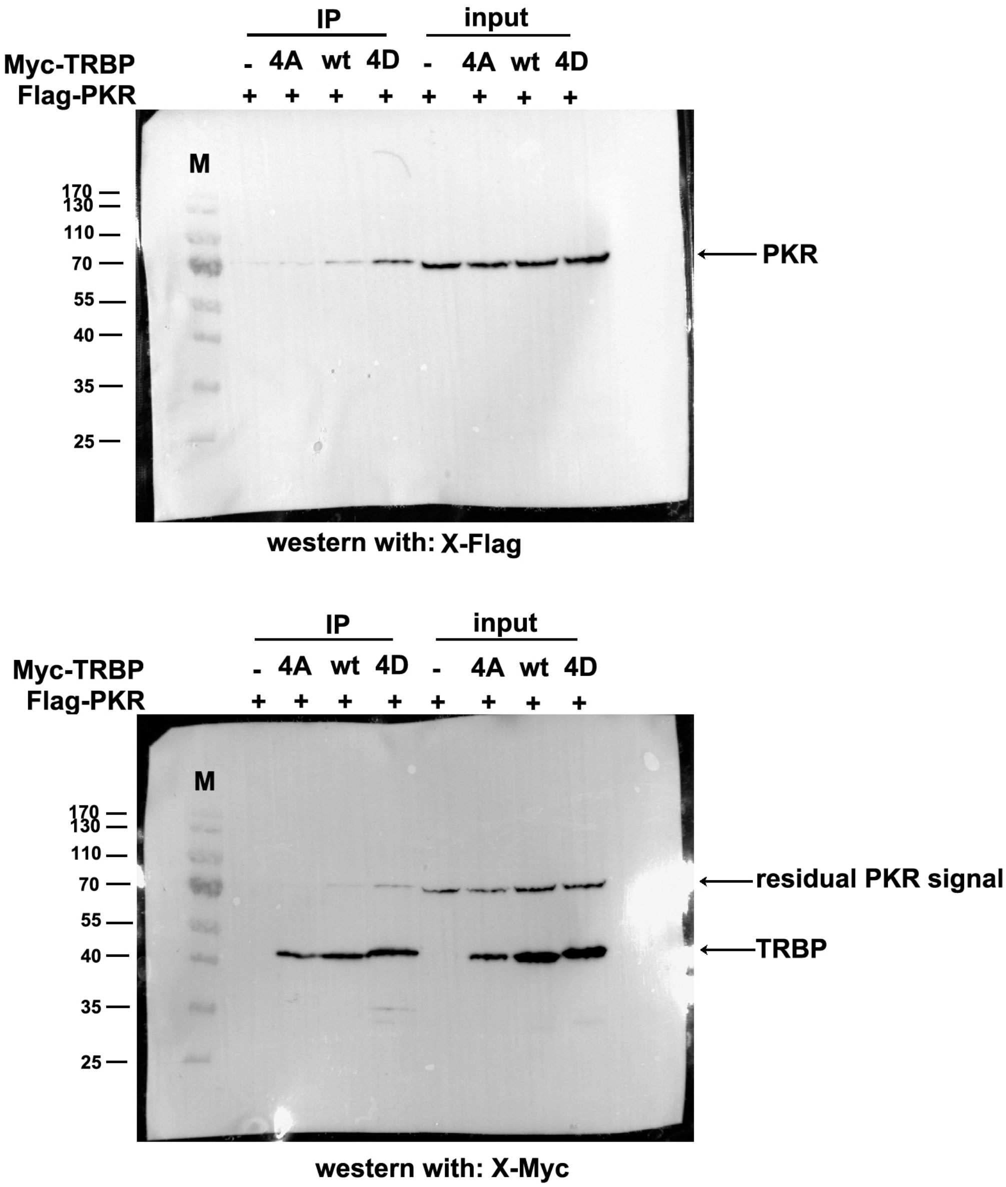
IP
input
Myc-TRBP
- 4A wt 4D - 4A wt 4D
Flag-PKR
+ + + + + + + +
M
170
130
110
70
55
40
35
25
PKR
western with: X-Flag
IP
input
Myc-TRBP
- 4A wt 4D - 4A wt 4D
Flag-PKR
+ + + + + + + +
M
170
130
110
70
55
40
35
25
residual PKR signal
TRBP
western with: X-Myc

Figure 5b

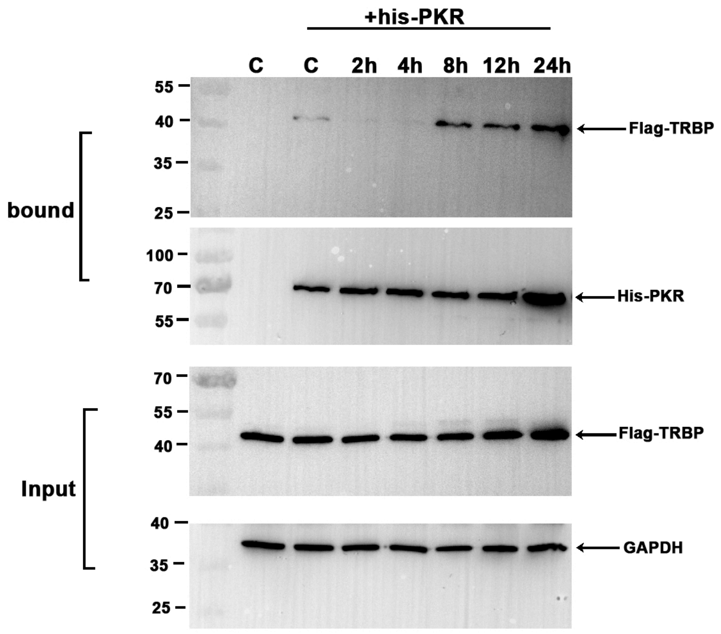
+his-PKR
C
C
2h
4h
8h
12h
24h
55
40
35
25
Flag-TRBP
bound
100
70
55
His-PKR
70
55
40
Flag-TRBP
Input
40
35
25
GAPDH

Figure 5c

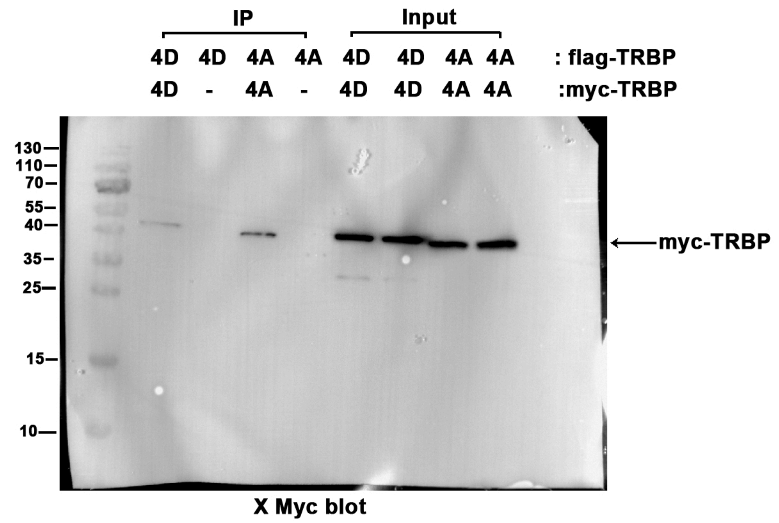
IP
Input
4D 4D 4A 4A 4D 4D 4A 4A : flag-TRBP
4D - 4A - 4D 4D 4A 4A :myc-TRBP
130
110
70
55
40
35
25
15
10
myc-TRBP
X Myc blot

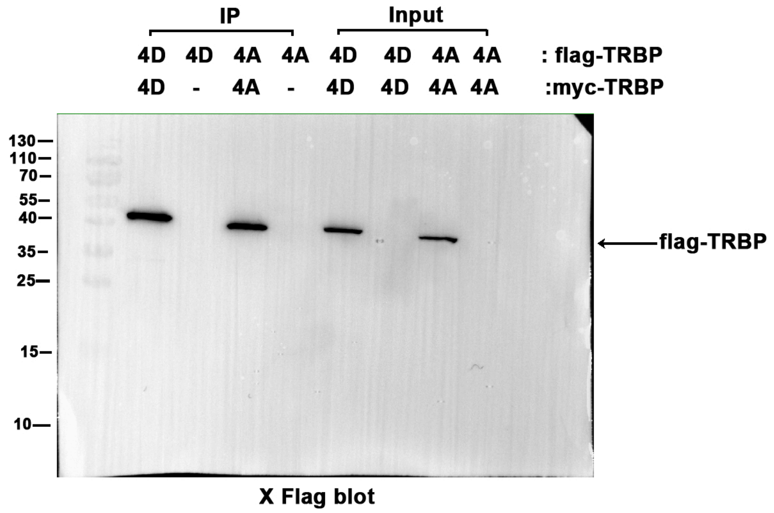
IP
Input
4D 4D 4A 4A 4D 4D 4A 4A : flag-TRBP
4D - 4A - 4D 4D 4A 4A :myc-TRBP
130
110
70
55
40
35
25
15
10
flag-TRBP
X Flag blot

Figure 5e

Supplementary Figure 1a: Full length western blots showing Flag TRBP induction after doxycycline removal in doxycycline inducible Flag TRBP overexpressing HeLa tet off cell line.

HeLa-Tet off (Clontech) cells were stably transfected with the FlagTRBP/pTRE2pur expression construct as described in Materials and Methods. To characterize the induction of the Flag TRBP expression construct in one selected clone, the cells were washed twice with 1X PBS, and lysates were collected at each indicated time point after the removal of doxycycline from the growth medium. 50 µg of total protein from each cell lysate was resolved on a 12% SDS-polyacrylamide gel and transferred to a PVDF membrane. The membrane was subsequently incubated in a mouse anti-Flag antibody conjugated to HRP, and the image was acquired using the LAS-7000 imaging system. The membrane was then incubated in a mouse-anti β-actin antibody conjugated to HRP and the image was acquired as above. Lanes in which the Precision Protein Standard ladder were loaded (M) were removed from the final figure shown in **Figure 1A.**

Supplementary Figure 1b: Full length agarose gel showing DNA Fragmentation in TRBP-HeLa cells compared to EV-HeLa cells after induction of stress with sodium arsenite.

The Flag TRBP overexpressing cells (TRBP-HeLa) described in Figure 1A were treated with 10 µM sodium arsenite at 48, 72, and 96 hours or left untreated. As a control, HeLa tet off cells stably expressing the pTRE2 pur expression construct (EV-HeLa) were subjected to similar treatment. Fragmented DNA from both sets of cells were collected as described in Materials and Methods, and resolved on a 1.5% agarose gel stained with ethidium bromide. Image was acquired using a Canon PowerShot S95 camera mounted on a UV transilluminator and converted to black and white with Photoshop. Areas of gel showing loading wells were removed from the final figure shown in **Figure 1B.** The blobs running at the bottom below 100 bp marker result from residual RNA left after RNase treatment and are usually only in untreated samples. These were cropped out from the final figure.

Supplementary Figure 1c: Western blots showing PARP cleavage in Flag TRBP overexpressing cells after treatment with sodium arsenite. TRBP-HeLa and EV-HeLa cells were treated with 25 μM sodium arsenite or left untreated and cell lysate was collected at each of the indicated time points. 50 μg of total protein was resolved on an 8% SDS-polyacrylamide gel and transferred to PVDF membranes. The membranes were cut just below the 55kDa marker; the top half of the membranes were incubated with a rabbit monoclonal antibody against PARP, while the bottom halves were incubated with a mouse monoclonal antibody against GAPDH conjugated to HRP. The top halves of each membrane were subsequently incubated with a goat anti-rabbit IgG HRP conjugated antibody. Images were acquired as described in Supplementary Figure 1A.

Supplementary Figure 2a: Full length western blot showing changes in TRBP's electrophoretic mobility in response to sodium-arsenite induced oxidative stress. TRBP-HeLa cells were treated with 10 μM sodium arsenite for the indicated time points or left untreated. Cell lysates were collected at each time point with phosphatase inhibitor cocktail, and 50 μg of total protein was resolved on a 12% SDS-polyacrylamide gel and transferred to a PVDF membrane. The membrane was incubated in a mouse anti-Flag antibody conjugated to HRP, and the image was acquired as previously described. The membrane was then incubated in a mouse-anti β-actin antibody conjugated to HRP and the image was acquired as above. Lanes in which the Precision Protein Standard ladder were loaded were removed from the final figure shown in Figure 2A. The 0.5h, 1h, 2h, 4h, 8h, 12h and 24h lanes in this figure were inverted such that they immediately followed the C (control) lane in that order in the final composite Figure 2a with Photoshop**.**

Supplementary Figure 2c: Full length western blot showing that changes in TRBP's electrophoretic mobility in response to sodium-arsenite induced oxidative stress are due to phosphorylation. TRBP-HeLa cells were treated with 10 μM sodium arsenite for the indicated time points or left untreated (control). Cell lysates were collected at each time point

with (*PPi* +) or without (*PPi* -) phosphatase inhibitor cocktail. 50 μg of total protein was resolved on a 12% SDS-polyacrylamide gel and transferred to a PVDF membrane – Prior to SDS-PAGE, 50 μg of total protein was subjected to phosphatase treatment (+*Ptase lanes only)*. The membrane was incubated in a mouse antibody against the flag epitope conjugated to HRP, and after image acquisition was incubated in a mouse antibody against β-actin conjugated to HRP. Image acquisition was as previously described.

Supplementary Figure 2d: Western blots showing ERK activation and phosphorylation of TRBP in response to sodium-arsenite induced oxidative stress. TRBP-HeLa cells were treated with 10 μM sodium arsenite and the MEK1/MEK2 inhibitor PD0325901 for the indicated time points or left untreated. Cell lysates were collected at each time point with phosphatase inhibitor cocktail, and 50 μg of total protein was resolved by SDS-PAGE and transferred to 2 PVDF membranes which were cut in half to separate the PD0325901 treated samples from the untreated samples. The first set of membranes were incubated in a mouse anti-Flag antibody conjugated to HRP, and the image was acquired as previously described. The second set of membranes were put through a succession of antibody incubations: first, in a rabbit antibody against phosphorylated Erk 1/2 and then a goat antibody against rabbit IgG, after which it was stripped after image acquisition. Second, the membranes were incubated in a rabbit antibody against Erk 1/2 and then a goat antibody against rabbit IgG after which an image was acquired. Finally, the membrane was incubated in a mouse-anti GAPDH antibody conjugated to HRP and the image was acquired as above.

Supplementary Figure 2e: Western blots showing JNK activation and phosphorylation of TRBP in response to sodium-arsenite induced oxidative stress. TRBP-HeLa cells were treated with 10 μM sodium arsenite and the JNK inhibitor SP600125 for the indicated time points or left untreated. Cell lysates were collected at each time point with phosphatase inhibitor cocktail, and 50 μg of total protein was resolved by SDS-PAGE and transferred to PVDF membranes which were cut in half to separate the inhibitor treated samples from the untreated

samples. The halved membranes were subsequently cut into strips between the 55 kDa and 35 kDa protein markers as we had only a limited amount of anti-p-JNK antibody. These were incubated in succession with mouse anti-Flag antibody, anti p-JNK, anti-JNK and anti-GAPDH. Flag and anti-GAPDH antibodies were conjugated to HRP and did not require a secondary antibody. Images were acquired as previously described. Lanes in which the Precision Protein Standard ladder were loaded were removed from the final figure panels shown in **Figure 2e.**

Supplementary Figure 5b: Full length western blots showing stronger interaction between phosphorylated TRBP and PKR as compared to unphosphorylated TRBP and PKR: HeLaM cells were transfected with the Flag-tagged K296R PKR expression construct alone or with myc-tagged expression constructs for wild type TRBP, the TRBP phospho-defective (TRBP AAAA) point mutant or the phospho-mimic (TRBP DDDD) point mutant. Total cell lysate (input), the immunoprecipitated myc-tagged TRBP proteins, and the co-immunoprecipitated Flag K296R PKR protein were resolved on a 10 % polyacrylamide gel and transferred to a PVDF membrane. The membrane was first incubated in a mouse antibody against the Flag epitope conjugated to HRP (x Flag blot). After image acquisition, the membrane was washed in 1X PBS and blocked in 5% milk in 1X PBS -Tween 20 for one hour before it was re-probed with a mouse antibody against the myc epitope. The lane (denoted 'M') in which the Precision Protein Standard ladder was loaded was excluded from the panels in **Figure 5b**.

Supplementary Figure 5c: Western blots showing changes in TRBP's association with PKR during sodium arsenite induced oxidative stress: Changes in TRBP's association with PKR during sodium arsenite induced oxidative stress was observed as described in Materials and Methods "TRBP-PKR pull-down assay". 25 μg of cell extract from TRBP-HeLa cells treated with 25 μM sodium arsenite for the indicated time points was incubated with 500 ng of recombinant His-tagged PKR immobilized on Ni^{2+} agarose beads. After three washes with immunoprecipitation buffer, the Ni^{2+}agarose beads associated proteins were resolved on an 8%

SDS polyacrylamide gel. 25 µg of cell extract was also resolved on another 8% gel to ensure that equal amounts of lysate were added for each sample (input). Proteins on both gels were transferred to two PVDF membranes. In the 'Bound' membrane, the membrane was incubated in a mouse antibody against the Flag epitope conjugated to HRP, and subsequently incubated in a mouse antibody against the His-epitope conjugated to HRP. In the 'Input' membrane, the membrane was incubated in a mouse antibody against the Flag epitope conjugated to HRP, and subsequently incubated in a mouse antibody against GAPDH conjugated to HRP. Images were acquired as previously described. Lanes in which the Precision Protein Standard ladder were loaded were removed from the final figure panels shown in **Figure 5c**.

Supplementary Figure 5e: Full length western blots showing stronger homomeric interaction between phosphorylated TRBP versus unphosphorylated TRBP: HeLaM cells were transfected with Flag and/or myc-tagged expression constructs for the TRBP phospho-defective (TRBP AAAA) or the phospho-mimic (TRBP DDDD) point mutants. Total cell lysate, immunoprecipitated Flag-TRBP AAAA/DDDD and co-immunoprecipitated myc-TRBP AAAA/DDDD proteins were resolved on 12% polyacrylamide gels, and transferred to a PVDF membrane. The membrane was first incubated in a mouse antibody against the myc epitope conjugated to HRP (x Myc blot), and after the image was acquired, was stripped and re-probed with a mouse antibody against the Flag epitope. The lane in which the Precision Protein Standard ladder was loaded was excluded from the final composite figure in **Figure 5e**.
